# Supplementary material for: Episodic evolution of coadapted sets of amino acid sites in mitochondrial proteins
Source: PLoS Genet. 2021 Jan 25;17(1):e1008711. doi: 10.1371/journal.pgen.1008711 (PMC7861529; doi:10.1371/journal.pgen.1008711)
Supplement: S1 Methods — Identifying interactions between sites. Visualizing contact and coevolution graphs. (PDF) [file pgen.1008711.s001.pdf]

Supplementary Methods

Inference of epistatic site pairs

The rooted phylogenetic tree with reconstructed genotypes for internal nodes is required for application of a phylogenetic method for predicting epistasis between pairs of sites. We assume that a substitution occurred on a branch of the tree at a certain site if ancestral and child nodes contain different alleles of this site; we imply that the substitution occurred in the middle of the branch. The key concept of the method is a concept of pairs of consecutive substitution. The two substitutions in a pair of sites on the phylogenetic tree are consecutive if 1) they both occur on one lineage and 2) no other substitutions at these sites occur between them on that lineage . To calculate epistatic statistics, firstly, we consider all ordered site pairs. For each pair of consecutive substitutions we calculated the exponential penalty for waiting time between the leading and the trailing substitution. The epistatic statistic for a site pair as defined in the original work of Kryazhimskiy et. al. 2011 [1] is the sum of such penalties. Here, we modified it so that for one leading substitution, the average penalty for all its trailing substitutions in the other site is taken into account. Thus, two pairs of substitutions with two different leading substitutions will have greater impact on the epistatic statistic than two pairs sharing a common leading substitution. We formally defined the new statistics as

$e_{(i,j)} = \sum_{\forall a \in B} \frac{\delta_i(a)}{\sum_{\forall b \in B(b \gtrsim a)} \delta_{i,j}(b \gtrsim a)} \sum_{\forall b \in B(b \gtrsim a)} \delta_{i,j}(b \gtrsim a)\, c_{i,j}(a,b)\, e^{-\frac{t_{a,b}}{\tau}}$, where $\boldsymbol{B}$ is a set of tree branches, the relation $b \gtrsim a$ denotes that the branch b coincides with the branch a or follows it on the tree, $\delta_i(a)$ and $\delta_{i,j}(b \gtrsim a)$ are indicator functions that take values 1 if corresponding conditions hold and 0 otherwise. The first condition requires that a substitution at the site *i* occurs on the branch *a.* The second condition additionally requires that a substitution at the site *j* occurs on the branch *b,* descendant to the branch *a.* $c_{i,j}(a,b)$ is a local probability of a pair of consecutive substitutions in sites *i, j* occurred on a pair of branches *a, b* and $t_{a,b}$ – the time spent between the substitutions, τ – is an average time between consecutive substitutions on the tree. For pairs of substitutions occurring on the same branch two possible variants of ordering are considered, thus, the local probability equals 0.5 for each variant of ordering. If two consecutive branches with no substitutions between them both contain substitutions at both sites, the local probability equals 0.25 for pairs of substitutions that occur on these branches. In this study we used epistatic statistics for unordered site pairs that is a sum of epistatic statistics of two ordered pairs: $e_{i,j} = e_{(i,j)} + e_{(j.i)}$.

The null model distribution of epistatic statistics is obtained by permutations of substitutions on the tree branches which preserve the number of substitutions for each site and for each branch. For that, we use BiRewire [2] package for R to reshuffle a substitution incidence matrix, which is a binary matrix where 1 means an occurrence of a substitution at a particular site on a particular branch and 0 means an absence of a substitution at a site on a branch. Matrix has a number of columns which equals to the number of sites and a number of rows which equals to the number of branches. We performed 10000 permutations in total. For each site pair an average value of epistatic statistic, variance and two tail probabilities (upper and lower p-values) are calculated. Low values of lower p-values correspond to deficit of consecutive pairs of substitutions in the site pair (***discordant evolution***), on the contrary, low values of upper p-values correspond to excess (***concordant evolution***).

To correct nominal p-values obtained for the data for multiple testing we estimated the false discovery (FDR) rates [3,4]. To estimate FDR we select 400 out of 10000 permutations referred further as fake dataset. For each selected permutation we calculated epistatic statistics and p-values. For each p-value threshold we calculate the corresponding number of findings for the real dataset (R – declared positives) and average number of findings in the fake dataset (E[V] – false positives). The FDR is a ratio of E[V] to R.

Identifying interactions between sites

Site pairs couldn't be directly compared by their epistatic statistics, and to make them comparable we used their z-score transformations: $z_{i,j} = \frac{e_{i,j} - m_{i,j}}{\sqrt{v_{i,j}}}$, where $m_{i,j}$ is mean and $v_{i,j}$ is variance. We refer to z-scores normalized on the maximal absolute z-score value as ***pseudo-correlations***. We set the pseudo-correlation for a site with itself equal to 1. Generally, pseudo-correlations for different pairs of sites are not independent of each other. Thus, we are interested in direct interactions, following the previous studies [5] we transform the positive pseudo-correlations into the partial correlation matrix with independent elements, using the cor2pcor R package (http://www.strimmerlab.org/software/corpcor/) [6].

To identify selective forces that directed evolution of different pairs of sites, for each gene we constructed coevolution graph where vertices were sites and branches were significantly concordantly or discordantly evolved site pairs. We define ***association statistics*** which we used to weight branches of coevolution graphs as follows. By definition, for concordantly evolved site pair association statistics equals to partial correlation if partial correlation is positive. Concordantly evolved site pairs having negative values of partial correlations

or insignificant upper p-values were not represented by edges on coevolution graphs. An upper p-value is insignificant if it is higher than the minimum of two values: 0.05 and a p-value threshold for FDR<0.3. For a discordantly evolved site pair, by definition, association statistics equals to pseudo-correlation. On the coevolution graph, discordantly evolving site pairs were represented by edges if their corresponding lower p-values are below 0.05.

Visualizing contact and coevolution graphs

To visualize interactions between coevolving groups of sites we built small graphs. For each gene these small graphs are compact representations of big graphs: the contact graph, the positive edge subgraph of the coevolution graph and negative edge subgraph of the coevolution graph. Big graphs contain all protein sites as vertices and edges connect two vertices if corresponding sites are close to each other in 3D structure (for the contact graph) or these sites evolve concordantly or discordantly (for the coevolution graph). In small graphs vertices represent groups of coevolving sites found by the modularity method for graphs with signed edge weights [7] applied for coevolution graphs. Arcs in small graphs represent relations between vertices in original graphs: an arc connecting a group with itself represents internal edges between vertices in this group (internal arc) and an arc connecting different groups represents intergroup edges (external arc). Two vertices v_1 and v_2 of a small graph are connected with an external arc if there is at least one external edge in the corresponding big graph between groups v_1 and v_2. Vertex is connected to itself if there is at least one edge in the big graph connecting vertices attributed into the corresponding group. We assigned weight for each external arc between vertices i and j :

$w_{i,j} = ln\left(n_{ij}\right) - ln\left(\frac{1}{N}\frac{n_i n_j}{2}\right)$, where n_{ij} is the number of edges (for representations of contact graphs) or total weight of the edges (for representations of coevolution graphs) between groups i and j in the big graph, n_k is the number of edges (weight of all edges) in group k, N is the total number of edges (total weight of all edges) in the big graph and $\frac{1}{N}\frac{n_i n_j}{2}$ is the expected number of edges between groups i and j according to the modularity model [7] with only difference that we divide by 2 since our edges are not directed.

References

1. Kryazhimskiy S, Dushoff J, Bazykin GA, Plotkin JB. Prevalence of epistasis in the evolution of influenza A surface proteins. PLoS Genet. 2011;7: e1001301. doi:10.1371/journal.pgen.1001301
2. Iorio F, Bernardo-Faura M, Gobbi A, Cokelaer T, Jurman G, Saez-Rodriguez J. Efficient randomization of biological networks while preserving functional characterization of individual nodes. BMC

Bioinformatics. 2016;17. doi:10.1186/s12859-016-1402-1

3. Genovese C, Wasserman L. A stochastic process approach to false discovery control. Ann Statist. 2004;32: 1035–1061. doi:10.1214/009053604000000283

4. Benjamini Y. Simultaneous and selective inference: Current successes and future challenges. Biom J. 2010;52: 708–721. doi:10.1002/bimj.200900299

5. Jones DT, Buchan DWA, Cozzetto D, Pontil M. PSICOV: precise structural contact prediction using sparse inverse covariance estimation on large multiple sequence alignments. Bioinformatics. 2012;28: 184–190. doi:10.1093/bioinformatics/btr638

6. Schäfer J, Strimmer K. A shrinkage approach to large-scale covariance matrix estimation and implications for functional genomics. Stat Appl Genet Mol Biol. 2005;4: Article32. Doi:10.2202/1544-6115.1175

7. Traag VA, Bruggeman J. Community detection in networks with positive and negative links. Physical Review E. 2009;80. doi:10.1103/PhysRevE.80.036115
