# Supplementary material for: Episodic evolution of coadapted sets of amino acid sites in mitochondrial proteins
Source: PLoS Genet. 2021 Jan 25;17(1):e1008711. doi: 10.1371/journal.pgen.1008711 (PMC7861529; doi:10.1371/journal.pgen.1008711)
Supplement: S1 Table — For each gene, the number of predicted site pairs (#pairs) and the nominal p-values corresponding to FDR<0.3 are shown. (DOCX) [file pgen.1008711.s002.docx]

Table S1. Numbers of concordantly (+) and discordantly (-) evolved site pairs predicted by the method with applied correction for phylogenetic uncertainty.

| gene | pairs, concordant (+) | #pairs | nominal p-value threshold for FDR<0.3 |
| --- | --- | --- | --- |
|  | discordant (-) |  |  |
| ATP6 |  | | |
|  | + | 1175 | 0.0146 |
|  | - | 5986 | 0.05 |
| CYTB |  | | |
|  | + | 1713 | 0.007 |
|  | - | 12259 | 0.05 |
| COX1 |  | | |
|  | + | 9004 | 0.231 |
|  | - | 12173 | 0.05 |
| COX2 |  | | |
|  | + | 450 | 0.0057 |
|  | - | 4241 | 0.05 |
| COX3 |  | | |
|  | + | 2176 | 0.021 |
|  | - | 4638 | 0.05 |

For each gene, the number of predicted site pairs (#pairs) and the nominal p-values corresponding to FDR<0.3 are shown.
