# Supplementary material for: Episodic evolution of coadapted sets of amino acid sites in mitochondrial proteins
Source: PLoS Genet. 2021 Jan 25;17(1):e1008711. doi: 10.1371/journal.pgen.1008711 (PMC7861529; doi:10.1371/journal.pgen.1008711)
Supplement: S2 Table — For concordantly evolving pairs, positive correlation of association statistics with clustering z-scores means that the excess of rapid consecutive substitutions is accompanied by more prominent “clustering” of non-consecutive substitutions. Similarly, for discordantly evolving pairs, positive correlation means that the deficit of rapid consecutive substitutions is accompanied by remoteness of non-consecutive ones. Thus, in both cases, positive correlation implies that episodic selection contributes to the association statistics. (DOCX) [file pgen.1008711.s003.docx]

Table S2. Correlations between strengths of clustering of non-consecutive substitutions (clustering z-scores) and the values of excess or deficit of rapid consecutive substitutions (association statistics).

| gene | pairs, concordant (+)  discordant (-) | rho (Spearman’s) | p-value rho |  |
| --- | --- | --- | --- | --- |
|  |  |  |  |  |
| ATP6 |  | | |  |
|  | + | 0.24 | 1.41E-24 |  |
|  | - | 0.08 | 1.24E-08 |  |
| CYTB |  | | |  |
|  | + | 0.14 | 2.81E-11 |  |
|  | - | 0.007 | 0.42 |  |
| COX1 |  | | |  |
|  | + | 0.09 | 2.95E-21 |  |
|  | - | -0.07 | 4.46E-15 |  |
| COX2 |  | | |  |
|  | + | 0.04 | 0.2 |  |
|  | - | -0.096 | 1.05E-08 |  |
| COX3 |  | | |  |
|  | + | 0.34 | 8.84E-94 |  |
|  | - | -0.09 | 3.72E-10 |  |

For concordantly evolving pairs, positive correlation of association statistics with clustering z-scores means that the excess of rapid consecutive substitutions is accompanied by more prominent “clustering” of non-consecutive substitutions. Similarly, for discordantly evolving pairs, positive correlation means that the deficit of rapid consecutive substitutions is accompanied by remoteness of non-consecutive ones. Thus, in both cases, positive correlation implies that episodic selection contributes to the association statistics.
