# Supplementary material for: Episodic evolution of coadapted sets of amino acid sites in mitochondrial proteins
Source: PLoS Genet. 2021 Jan 25;17(1):e1008711. doi: 10.1371/journal.pgen.1008711 (PMC7861529; doi:10.1371/journal.pgen.1008711)
Supplement: S3 Table — For each protein the following statistics are shown: the numbers of significantly concordantly ('+') and discordantly ('-') evolving site pairs with known distances between sites in protein structures, those nominal p-values were below thresholds corresponding to FDR<0.3 (#pairs). The strength of excess (deficit) of consecutive substitutions is measured by two statistics—the pseudo-correlation and partial correlations (type of statistics). For concordantly evolved site pairs the association statistics equal to partial correlations and for discordantly evolved site pairs they equal to pseudo-correlations. The Spearman's correlations (rho) between distances on the protein structures and association statistics as well as corresponding p-values (rho p-value) are shown. The Spearman's rho between 3D distances and pseudo-correlations for concordantly evolved are also provided. For concordantly evolving pairs of sites, a significantly negative value of rho means that strongly associated sites tend to be closer on the structure; for discordantly evolving pairs of sites, a significantly negative rho means that strongly associated sites tend to be apart from each other. Some data from this table are presented in Table 3 in the main text of the manuscript. (DOCX) [file pgen.1008711.s004.docx]

Table S3. Numbers of concordantly and discordantly evolving site pairs mappable to the protein structures, and correlations between strength of excess (deficit) of rapid consecutive substitutions and distances between sites on protein structures.

| gene | concordant (+) | nominal p-value threshold for FDR<0.3 | #pairs | type of statistics | rho (Spearman’s) association statistics vs. 3D distances | rho p-value association statistics vs. 3D distances |
| --- | --- | --- | --- | --- | --- | --- |
|  | discordant (-) |  |  |  |  |  |
| ATP6 |  | | | | | |
|  | + | 0.0262 | 1764 | pseudo-correlation | -0.21 | <2.2e-16 |
|  |  |  |  | partial correlation | -0.15 | 6.96E-10 |
|  | - | 0.05 | 5213 | pseudo-correlation | -0.01 | 0.4795 |
| CYTB |  | | | | | |
|  | + | 0.0099 | 2216 | pseudo-correlation | -0.27 | <2.2e-16 |
|  |  |  |  | partial correlation | -0.24 | <2.2e-16 |
|  | - | 0.05 | 12782 | pseudo-correlation | -0.01 | 0.2759 |
| COX1 |  | | | | | |
|  | + | 0.0316 | 12114 | pseudo-correlation | -0.22 | <2.2e-16 |
|  |  |  |  | partial correlation | -0.24 | <2.2e-16 |
|  | - | 0.05 | 11431 | pseudo-correlation | -0.004 | 0.63 |
| COX2 |  | | | | | |
|  | + | 0.0135 | 875 | pseudo-correlation | -0.19 | 1.59E-08 |
|  |  |  |  | partial correlation | -0.24 | 8.64E-13 |
|  | - | 0.05 | 3522 | pseudo-correlation | -0.07 | 6.37E-05 |
| COX3 |  | | | | | |
|  | + | 0.0368 | 3375 | pseudo-correlation | -0.21 | <2.2e-16 |
|  |  |  |  | partial correlation | -0.16 | <2.2e-16 |
|  | - | 0.05 | 4637 | pseudo-correlation | -0.07 | 6.18E-06 |

For each protein the following statistics are shown: the numbers of significantly concordantly ('+') and discordantly ('-') evolving site pairs with known distances between sites in protein structures, those nominal p-values were below thresholds corresponding to FDR<0.3 (#pairs). The strength of excess (deficit) of consecutive substitutions is measured by two statistics - the pseudo-correlation and partial correlations (type of statistics). For concordantly evolved site pairs the association statistics equal to partial correlations and for discordantly evolved site pairs they equal to pseudo-correlations. The Spearman's correlations (rho) between distances on the protein structures and association statistics as well as corresponding p-values (rho p-value) are shown. The Spearman's rho between 3D distances and pseudo-correlations for concordantly evolved are also provided. For concordantly evolving pairs of sites, a significantly negative value of rho means that strongly associated sites tend to be closer on the structure; for discordantly evolving pairs of sites, a significantly negative rho means that strongly associated sites tend to be apart from each other. Some data from this table are presented in table 3 in the main text of the manuscript.
