# Supplementary material for: Episodic evolution of coadapted sets of amino acid sites in mitochondrial proteins
Source: PLoS Genet. 2021 Jan 25;17(1):e1008711. doi: 10.1371/journal.pgen.1008711 (PMC7861529; doi:10.1371/journal.pgen.1008711)
Supplement: S4 Table — (DOCX) [file pgen.1008711.s005.docx]

Table S4. Comparison of distances on protein structures for concordantly and discordantly evolved site pairs

| gene | mean pdb distance for concordant pairs, A | Std. dev. for concordant pairs | mean pdb distance for discordant pairs, A | Std. dev. for discordant pairs | Mann Whitney, P |
| --- | --- | --- | --- | --- | --- |
| ATP6 | 17.2 | 11.1 | 24.7 | 11.2 | < 2.2e-16 |
| CYTB | 17.6 | 12.1 | 27.5 | 11.4 | < 2.2e-16 |
| COX1 | 21.3 | 12.4 | 29.8 | 11.7 | < 2.2e-16 |
| COX2 | 19.6 | 15.3 | 29.1 | 16.5 | < 2.2e-16 |
| COX3 | 20.7 | 12.8 | 26.7 | 12.3 | < 2.2e-16 |
