# Supplementary material for: Episodic evolution of coadapted sets of amino acid sites in mitochondrial proteins
Source: PLoS Genet. 2021 Jan 25;17(1):e1008711. doi: 10.1371/journal.pgen.1008711 (PMC7861529; doi:10.1371/journal.pgen.1008711)
Supplement: S5 Table — The concordantly evolving site pairs are enriched by pairs of sites that are in contact on protein structures, oppositely, discordantly evolving site pairs are depleted by contacting site pairs. For each protein the following statistics are shown: the numbers of significantly concordantly ('+') and discordantly ('-') evolving site pairs with known distances between sites in protein structures, those nominal p-values were below thresholds corresponding to FDR<0.3 (#pairs), the number of contacting site pairs among the predicted concordantly and discordantly evolved pairs (#contacts) and proportions of total numbers of contacting pairs of site to total numbers of analysed site pairs (#all contacting pairs/#all pairs). Two sites are considered to be in contact on a corresponding protein structure if the minimal distance between heavy atoms of their side residuals is below 4A threshold. (DOCX) [file pgen.1008711.s006.docx]

Table S5. Numbers of contacting pairs among concordantly and discordantly evolving site pairs. The concordantly evolving site pairs are enriched by pairs of sites that are in contact on protein structures, oppositely, discordantly evolving site pairs are depleted by contacting site pairs.

| gene | concordant (+) | #pairs | #contacts | #all contacting pairs/#all pairs |
| --- | --- | --- | --- | --- |
|  | discordant (-) |  |  |  |
| ATP6 |  | | | |
|  | + | 1764 | 212 | 660/20100 |
|  | - | 5213 | 88 |  |
| CYTB |  | | | |
|  | + | 2216 | 301 | 1605/71253 |
|  | - | 12782 | 136 |  |
| COX1 |  | | | |
|  | + | 12114 | 939 | 2045/112101 |
|  | - | 11431 | 49 |  |
| COX2 |  | | | |
|  | + | 875 | 129 | 700/20910 |
|  | - | 3522 | 49 |  |
| COX3 |  | | | |
|  | + | 3375 | 312 | 998/29646 |
|  | - | 4637 | 82 |  |

For each protein the following statistics are shown: the numbers of significantly concordantly ('+') and discordantly ('-') evolving site pairs with known distances between sites in protein structures, those nominal p-values were below thresholds corresponding to FDR<0.3 (#pairs), the number of contacting site pairs among the predicted concordantly and discordantly evolved pairs (#contacts) and proportions of total numbers of contacting pairs of site to total numbers of analysed site pairs (#all contacting pairs/#all pairs). Two sites are considered to be in contact on a corresponding protein structure if the minimal distance between heavy atoms of their side residuals is below 4A threshold.
