# Supplementary material for: Episodic evolution of coadapted sets of amino acid sites in mitochondrial proteins
Source: PLoS Genet. 2021 Jan 25;17(1):e1008711. doi: 10.1371/journal.pgen.1008711 (PMC7861529; doi:10.1371/journal.pgen.1008711)
Supplement: S6 Table — Higher values of MAP correspond to stronger dependencies of amino acid substitution probabilities in one site on the background amino acid in another site in a pair. (DOCX) [file pgen.1008711.s007.docx]

Table S6. Mutual allele preference statistics (MAP) are higher for concordantly evolved site pairs than for other site pairs

| gene | Mean MAP for concordant pairs | MAP std. dev. for concordant pairs | Mean MAP for other pairs | MAP std. dev. of other pairs | Mann Whitney test, P |
| --- | --- | --- | --- | --- | --- |
| ATP6 | 0.71 | 0.12 | 0.70 | 0.10 | 0.002 |
| CYTB | 0.73 | 0.17 | 0.68 | 0.14 | 2.22E-33 |
| COX1 | 0.75 | 0.17 | 0.70 | 0.16 | 2.27E-93 |
| COX2 | 0.71 | 0.15 | 0.68 | 0.15 | 2.46E-06 |
| COX3 | 0.68 | 0.15 | 0.68 | 0.14 | 0.59 |

Higher values of MAP correspond to stronger dependencies of amino acid substitution probabilities in one site on the background amino acid in another site in a pair.
