# Supplementary material for: Episodic evolution of coadapted sets of amino acid sites in mitochondrial proteins
Source: PLoS Genet. 2021 Jan 25;17(1):e1008711. doi: 10.1371/journal.pgen.1008711 (PMC7861529; doi:10.1371/journal.pgen.1008711)
Supplement: S7 Table — (DOCX) [file pgen.1008711.s008.docx]

Table S7. Correlations of the mutual allele preference statistic (MAP) and association statistics for concordantly evolving site pairs.

| gene | rho (Spearman's), association statistics vs. MAP | P-val., rho |
| --- | --- | --- |
| ATP6 | 0.06 | 0.008 |
| CYTB | 0.12 | 9.33E-08 |
| COX1 | 0.09 | 1.00E-15 |
| COX2 | 0.13 | 0.0003 |
| COX3 | 0.13 | 4.62E-12 |
