# Supplementary material for: Episodic evolution of coadapted sets of amino acid sites in mitochondrial proteins
Source: PLoS Genet. 2021 Jan 25;17(1):e1008711. doi: 10.1371/journal.pgen.1008711 (PMC7861529; doi:10.1371/journal.pgen.1008711)
Supplement: S8 Table — Among concordantly evolving site pairs the pairs of sites proximal on protein structures also have higher values of MAP statistic than pairs of distant sites. Mean values of MAP for contacting and non-contacting on protein structures concordantly evolved site pairs their standard deviations and p-values of Mann Whitney U-test are shown. The Spearman’s correlation (rho) of MAPs and distances on protein structures and probabilities that observed correlations equal to zero (P-val., rho) are also provided. (DOCX) [file pgen.1008711.s009.docx]

Table S8. Comparison of the mutual allele preference statistic (MAP) for proximal and distant concordantly evolving site pairs.

| gene | Mean MAP for contact pairs | MAP std. dev. for contact pairs | Mean MAP for non-contact pairs | MAP std. dev. for non-contact pairs | Mann Whitney test, P | rho, 3D distance vs. MAP | P-val., rho |
| --- | --- | --- | --- | --- | --- | --- | --- |
| ATP6 | 0.74 | 0.12 | 0.70 | 0.12 | 7.52E-06 | -0.16 | 3.30E-11 |
| CYTB | 0.78 | 0.16 | 0.73 | 0.17 | 6.88E-06 | -0.17 | 3.37E-15 |
| COX1 | 0.81 | 0.16 | 0.74 | 0.17 | 1.14E-27 | -0.17 | 5.38E-57 |
| COX2 | 0.76 | 0.13 | 0.71 | 0.16 | 1.82E-05 | -0.21 | 6.57E-09 |
| COX3 | 0.74 | 0.16 | 0.68 | 0.15 | 2.36E-09 | -0.15 | 2.76E-16 |

Among concordantly evolving site pairs the pairs of sites proximal on protein structures also have higher values of MAP statistic than pairs of distant sites. Mean values of MAP for contacting and non-contacting on protein structures concordantly evolved site pairs their standard deviations and p-values of Mann Whitney U-test are shown.The Spearman’s correlation (rho) of MAPs and distances on protein structures and probabilities that observed correlations equal to zero (P-val., rho) are also provided.
