# Supplementary material for: Episodic evolution of coadapted sets of amino acid sites in mitochondrial proteins
Source: PLoS Genet. 2021 Jan 25;17(1):e1008711. doi: 10.1371/journal.pgen.1008711 (PMC7861529; doi:10.1371/journal.pgen.1008711)
Supplement: S9 Table — Low absolute values of the substitution clustering z-score statistic correspond to high values of mutual allele preference. The Spearman’s correlation (rho) of MAPs and absolute values of substitution clustering z-scores and probabilities that observed correlations equal to zero (P-val., rho) are shown. (DOCX) [file pgen.1008711.s010.docx]

Table S9. Correlation between absolute values of clustering z-score and the mutual allele preference statistic (MAP) for significantly concordantly coevolving site pairs.

| gene | rho (Spearman's), abs. clustering z-score vs. MAP | P-val., rho |
| --- | --- | --- |
| ATP6 | -0.18 | 7.73E-15 |
| CYTB | -0.25 | 3.37E-29 |
| COX1 | -0.19 | 7.83E-73 |
| COX2 | -0.20 | 9.67E-09 |
| COX3 | -0.10 | 7.24E-08 |

Low absolute values of the substitution clustering z-score statistic correspond to high values of mutual allele preference. The Spearman’s correlation (rho) of MAPs and absolute values of substitution clustering z-scores and probabilities that observed correlations equal to zero (P-val., rho) are shown.
