# Supplementary material for: Episodic evolution of coadapted sets of amino acid sites in mitochondrial proteins
Source: PLoS Genet. 2021 Jan 25;17(1):e1008711. doi: 10.1371/journal.pgen.1008711 (PMC7861529; doi:10.1371/journal.pgen.1008711)
Supplement: S10 Table — For each protein the number of analysed sites of a corresponding multiple alignment (#sites), the number of vertices in a coevolution graph (#vertices) and numbers of edges with positive (#positive edges) and negative (#negative edges) weights are shown. (DOCX) [file pgen.1008711.s011.docx]

Table S10. Characteristics of coevolution graphs.

| gene | #sites | #vertices | #positive edges | #negative edges |
| --- | --- | --- | --- | --- |
| ATP6 | 221 | 213 | 2106 | 5511 |
| CYTB | 386 | 373 | 2452 | 12187 |
| COX1 | 485 | 481 | 12309 | 12721 |
| COX2 | 220 | 210 | 1086 | 4122 |
| COX3 | 250 | 241 | 3809 | 3738 |

For each protein the number of analysed sites of a corresponding multiple alignment (#sites), the number of vertices in a coevolution graph (#vertices) and numbers of edges with positive (#positive edges) and negative (#negative edges) weights are shown.
