# Supplementary material for: Episodic evolution of coadapted sets of amino acid sites in mitochondrial proteins
Source: PLoS Genet. 2021 Jan 25;17(1):e1008711. doi: 10.1371/journal.pgen.1008711 (PMC7861529; doi:10.1371/journal.pgen.1008711)
Supplement: S12 Table — See S11 Table for detailed description. (DOCX) [file pgen.1008711.s013.docx]

Table S12. Coevolution of surface sites of COX2 and interactions with other proteins of the respiratory complex IV.

| cox2 contacts with other mitochondrially-encoded subunits of COX |  |  | | | | | |
| --- | --- | --- | --- | --- | --- | --- | --- |
|  | group | 1 | 2 | 3 | 4 | 5 | 6 |
| obs | NON CONT | 30 | 14 | 30 | 10 | 8 | 8 |
|  | CONT | 15 | 11 | 3 | 16 | 12 | 1 |
|  | sum | 45 | 25 | 33 | 26 | 20 | 9 |
| exp | NON CONT | 25.7 | 13.7 | 24.6 | 20.7 | 9.2 | 7.3 |
|  | CONT | 19.3 | 11.3 | 8.4 | 5.3 | 10.8 | 1.7 |
|  | sum | 45 | 25 | 33 | 26 | 20 | 9 |
|  | group upper pvalue | 0.9229 | 0.6373 | 0.9978 | 0.0026 | 0.4387 | 0.6927 |
|  | group lower pvalue | 0.1231 | 0.5184 | 0.0123 | 0.9987 | 0.7151 | 0.5522 |
|  | table p_value = 0.0056 |  | | | | | |
|  | | | | | | | |
| cox2 contacts with nuclearly-encoded subunits of COX |  |  | | | | | |
|  | group | 1 | 2 | 3 | 4 | 5 | 6 |
| obs | NON CONT | 22 | 15 | 17 | 17 | 17 | 5 |
|  | CONT | 23 | 10 | 16 | 9 | 3 | 4 |
|  | sum | 45 | 25 | 33 | 26 | 20 | 9 |
| exp | NON CONT | 24.1 | 13 | 22.1 | 19.4 | 9.5 | 6.9 |
|  | CONT | 20.9 | 12 | 10.9 | 6.6 | 10.5 | 2.1 |
|  | sum | 45 | 25 | 33 | 26 | 20 | 9 |
|  | group upper pvalue | 0.3537 | 0.8401 | 0.0727 | 0.2228 | 0.9954 | 0.2326 |
|  | group lower pvalue | 0.7624 | 0.2649 | 0.958 | 0.8297 | 0.0123 | 0.8651 |
|  | table p_value = 0.0877 |  | | | | | |
|  | | | | | | | |
| cox2 interface with others |  |  | | | | | |
|  | group | 1 | 2 | 3 | 4 | 5 | 6 |
| obs | ENC_noninterface | 5 | 4 | 11 | 1 | 3 | 4 |
|  | CONT + ENC_interface | 40 | 21 | 22 | 25 | 17 | 5 |
|  | sum | 45 | 25 | 33 | 26 | 20 | 9 |
| exp | ENC_noninterface | 8.7 | 4.7 | 7.1 | 5.1 | 4.3 | 1.7 |
|  | CONT + ENC_interface | 36.3 | 20.3 | 25.9 | 20.9 | 15.7 | 7.3 |
|  | sum | 45 | 25 | 33 | 26 | 20 | 9 |
|  | group upper pvalue | 0.0787 | 0.472 | 0.9737 | 0.0221 | 0.3446 | 0.9851 |
|  | group lower pvalue | 0.9715 | 0.7331 | 0.0644 | 0.9966 | 0.8339 | 0.0681 |
|  | table p_value =0.0282 |  | | | | | |

The protein surface sites, sites contacting with other subunits of the complex and noncontact interface sites are identified as described in Methods. The following definitions of three sets of sites participating in interactions with other subunits of the protein complex are considered: (i) sites forming direct contacts (CONT) with other mitochondrially-encoded subunits of COX, (ii) sites forming direct contacts (CONT) with nuclearly-encoded subunits and (iii) the union of sites forming contacts with any other subunit and noncontact interface sites (CONT + ENC_interface). The complementary subsets of sites are noncontact surface sites (NON CONTACT) and surface noncontact noninterface sites (ENC_noninterface). For each set of interacting sites (i-iii) two contingency tables with distributions of observed and expected counts of sites in coevolving groups are shown. The expected counts were obtained by sampling random subgraphs having the same or higher density of edges as were in subgraphs of the contact graph which correspond to the sets (i-iii) of interacting sites. For each group of coevolving sites the Jaccard-index is used as a measure of its overlap with sets of interacting sites (i-iii), for each set two p-values were calculated: the fraction of samples having the same or greater statistics as observed (upper p-value) and the fraction of samples having the same or smaller statistics (lower value). Low values of the upper (lower) p-values correspond to enrichment (avoidance) of sites addressed into each group of coevolving sites among sites interacting with other subunits (i-iii). For each set of interacting sites (i-iii), the hi^2 statistic is used as a measure of deviations of observed site counts from expected ones for all groups together, for which a "table p-value" is calculated.
