# Supplementary material for: Episodic evolution of coadapted sets of amino acid sites in mitochondrial proteins
Source: PLoS Genet. 2021 Jan 25;17(1):e1008711. doi: 10.1371/journal.pgen.1008711 (PMC7861529; doi:10.1371/journal.pgen.1008711)
Supplement: S16 Table — Each protein has changed substitution rates in groups of coevolving sites several times during evolution, such changes are superimposed on the phylogeny of mitochondrial proteins. For each protein the following statistics are shown: (i) the number of branches on the tree to which episodes of changes of substitution rates have been assigned, for some of these branches the assignment may be ambiguous (see Methods), (ii) a number of these branches for which all five proteins accumulated enough substitutions to test the concordance of changes and (iii) the number of parental branches of (ii) which could be unambiguously used as identifiers of episodes of changes of substitution rates for testing for concordance. (DOCX) [file pgen.1008711.s017.docx]

Table S16. Substitutions rates in groups of coevolving sites have changed during evolution of Metazoa and Fungi.

| Protein | ATP6 | COX1 | COX2 | COX3 | CYTB |
| --- | --- | --- | --- | --- | --- |
| (i) no. of branches (internals) | 66 | 83 | 41 | 49 | 106 |
| (ii) no. of branches (internals with enough mutations to apply tests for all proteins) | 56 | 75 | 40 | 47 | 99 |
| (iii) no. of parental branches (internals with enough mutations to apply tests for all proteins) | 42 | 53 | 28 | 32 | 62 |

Each protein has changed substitution rates in groups of coevolving sites several times during evolution, such changes are superimposed on the phylogeny of mitochondrial proteins. For each protein the following statistics are shown: (i) the number of branches on the tree to which episodes of changes of substitution rates have been assigned, for some of these branches the assignment may be ambiguous (see Methods), (ii) a number of these branches for which all five proteins accumulated enough substitutions to test the concordance of changes and (iii) the number of parental branches of (ii) which could be unambiguously used as identifiers of episodes of changes of substitution rates for testing for concordance.
