# Supplementary figures and images for: Episodic evolution of coadapted sets of amino acid sites in mitochondrial proteins
Source: PLoS Genet. 2021 Jan 25;17(1):e1008711. doi: 10.1371/journal.pgen.1008711 (PMC7861529; doi:10.1371/journal.pgen.1008711)

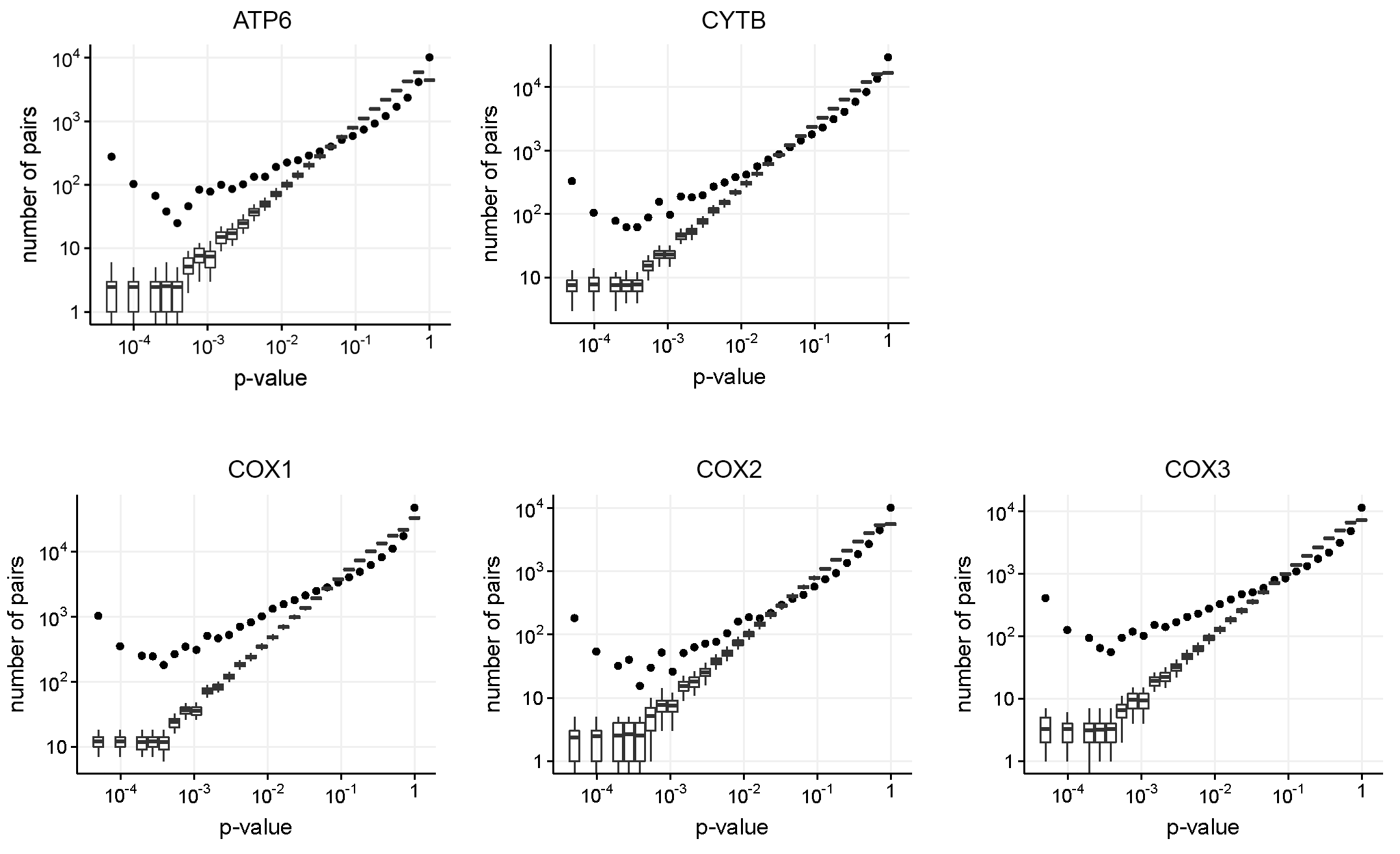

Supplement: S1 Fig — Black dots indicate data points, boxes with whiskers indicate simulation results. Top and bottom of each box correspond to the 75th and 25th percentile, whiskers correspond to the 95th and 5th percentile. (TIF) [file pgen.1008711.s018.tif]

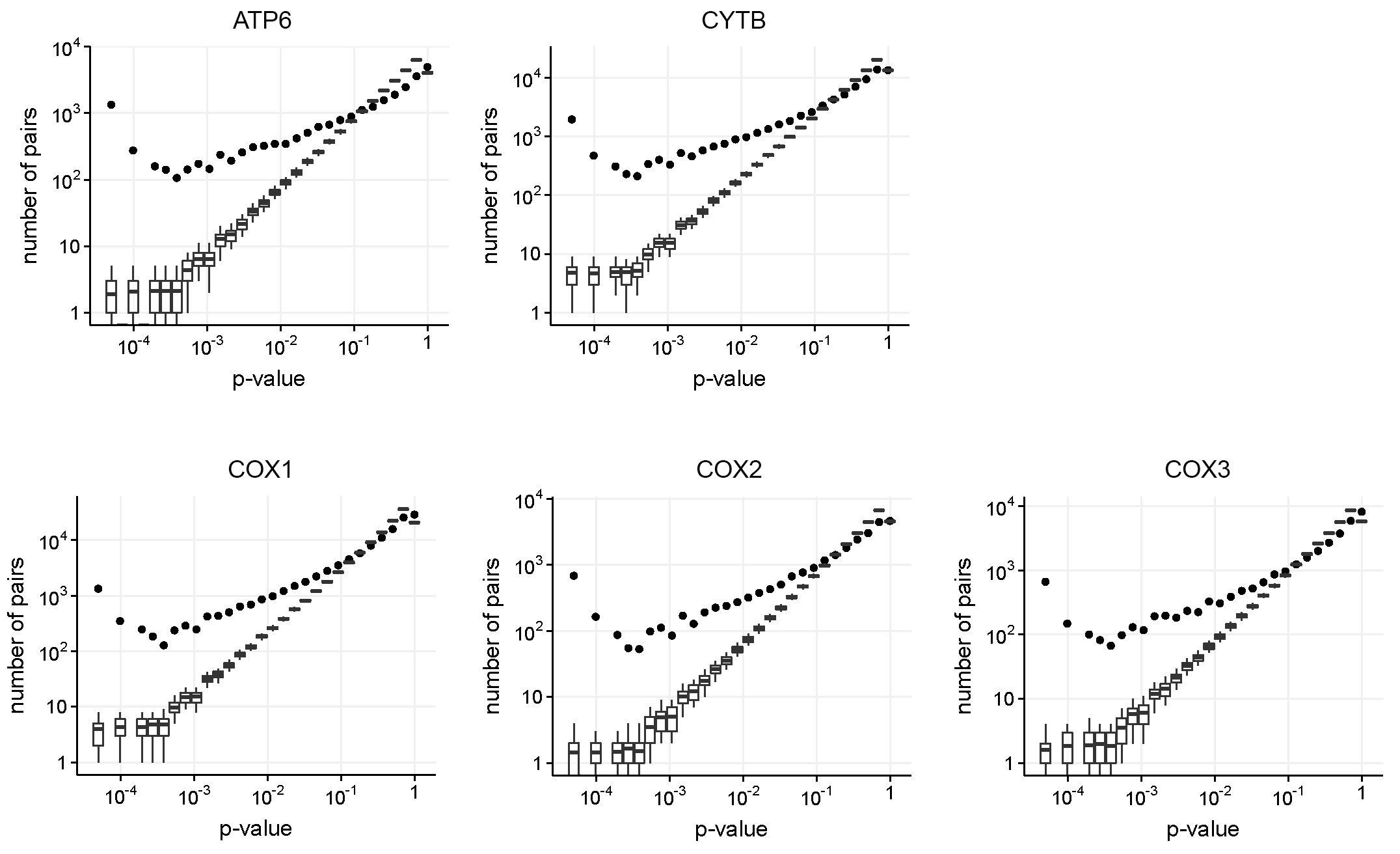

Supplement: S2 Fig — Black dots indicate data points, boxes with whiskers indicate simulation results. Top and bottom of each box correspond to the 75th and 25th percentile, whiskers correspond to the 95th and 5th percentile. (TIF) [file pgen.1008711.s019.tif]

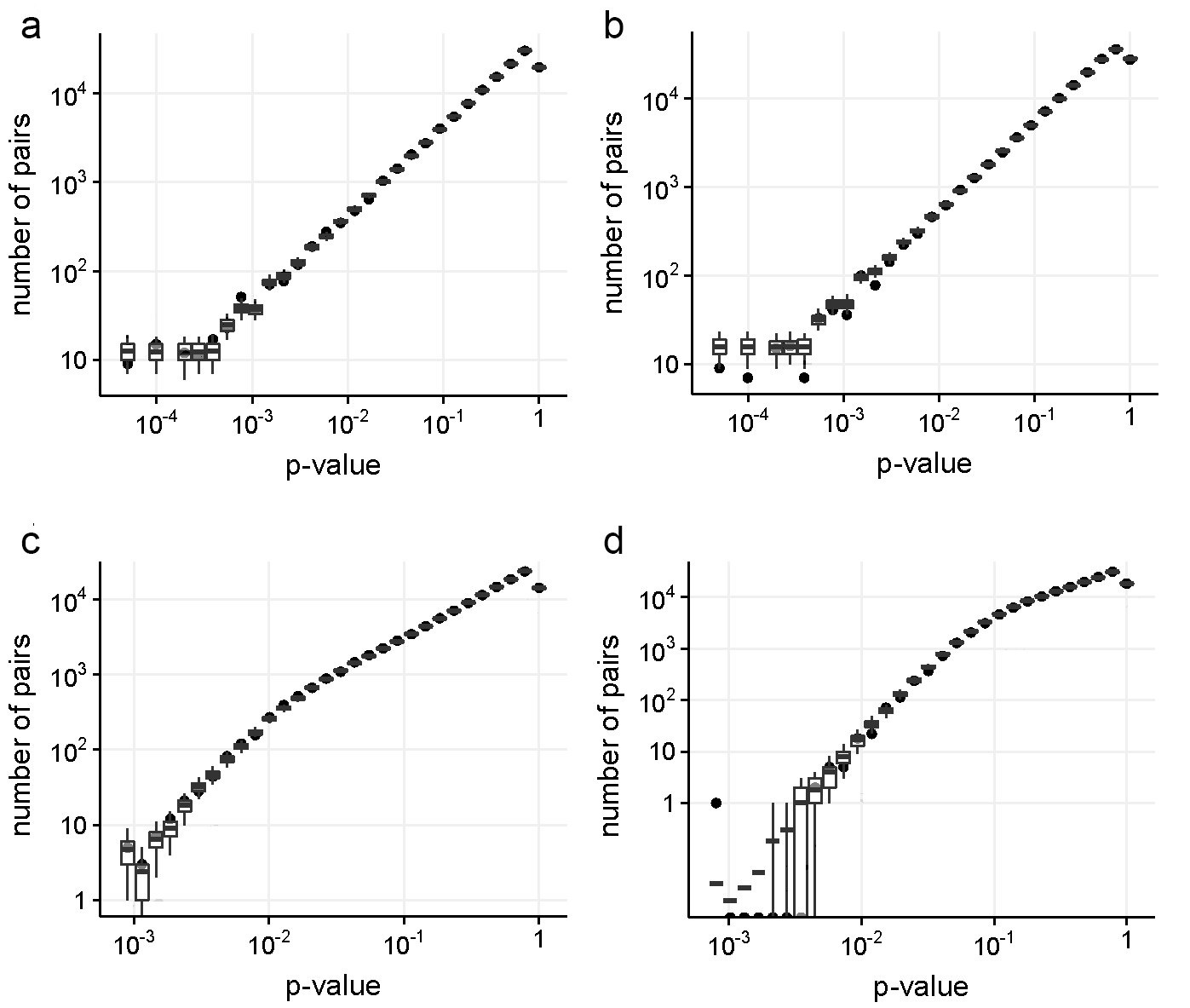

Supplement: S3 Fig — a,c—episodic positive selection, b,d—hitchhiking. Black dots indicate data points, boxes with whiskers indicate simulation results. Top and bottom of each box correspond to the 75th and 25th percentile, whiskers correspond to the 95th and 5th percentile. (TIF) [file pgen.1008711.s020.tif]

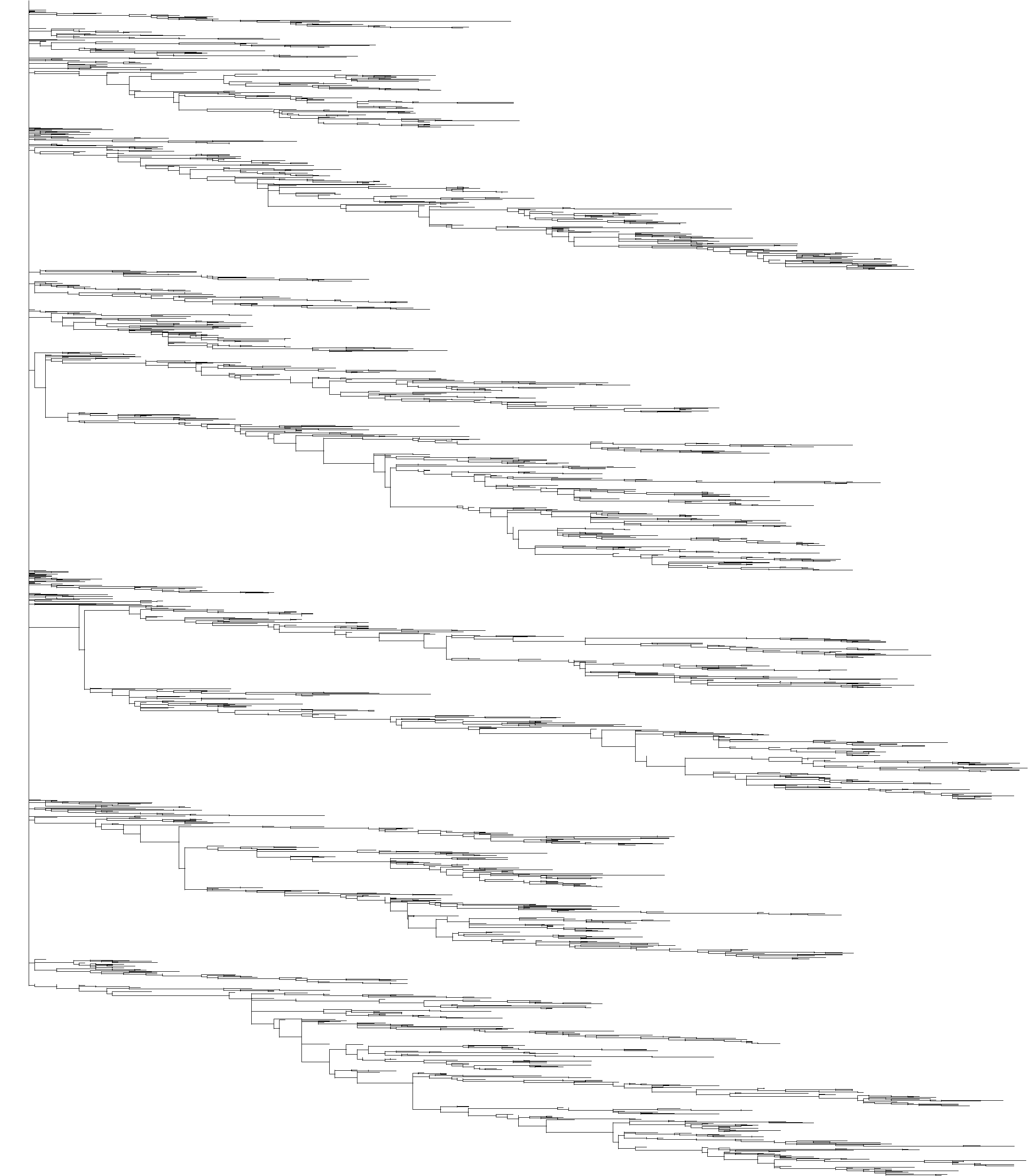

Supplement: S4 Fig — Since initial genotypes contain many sites with non-optimal amino acids, there are multiple available ways for adaptation, which lead to coexistence of multiple long living clades and causes clonal interference. (TIF) [file pgen.1008711.s021.tif]

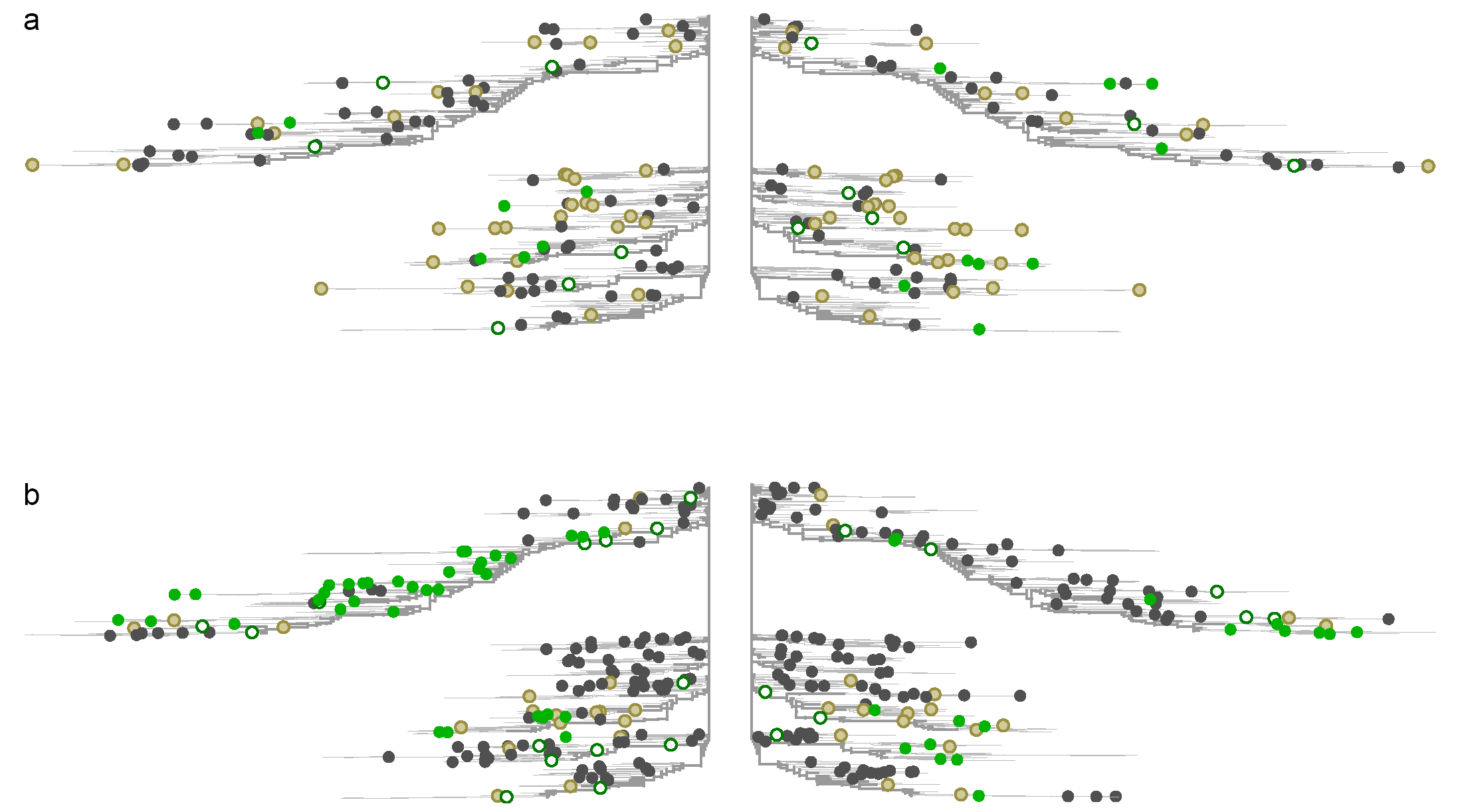

Supplement: S5 Fig — Illustrative pairs of sites which evolved under positive (a) or negative (b) epistasis during the forward simulation of evolution. Under positive epistasis, substitutions which were followed (empty green circles) or preceded (green dots) by a substitution at the other site, as well as same-branch substitutions and substitutions which have both leading and trailing counterparts (beige dots) are overrepresented. On the contrary, under negative epistasis, substitutions without immediate leading or trailing counterparts at the other site (black dots) are overrepresented. (TIF) [file pgen.1008711.s022.tif]

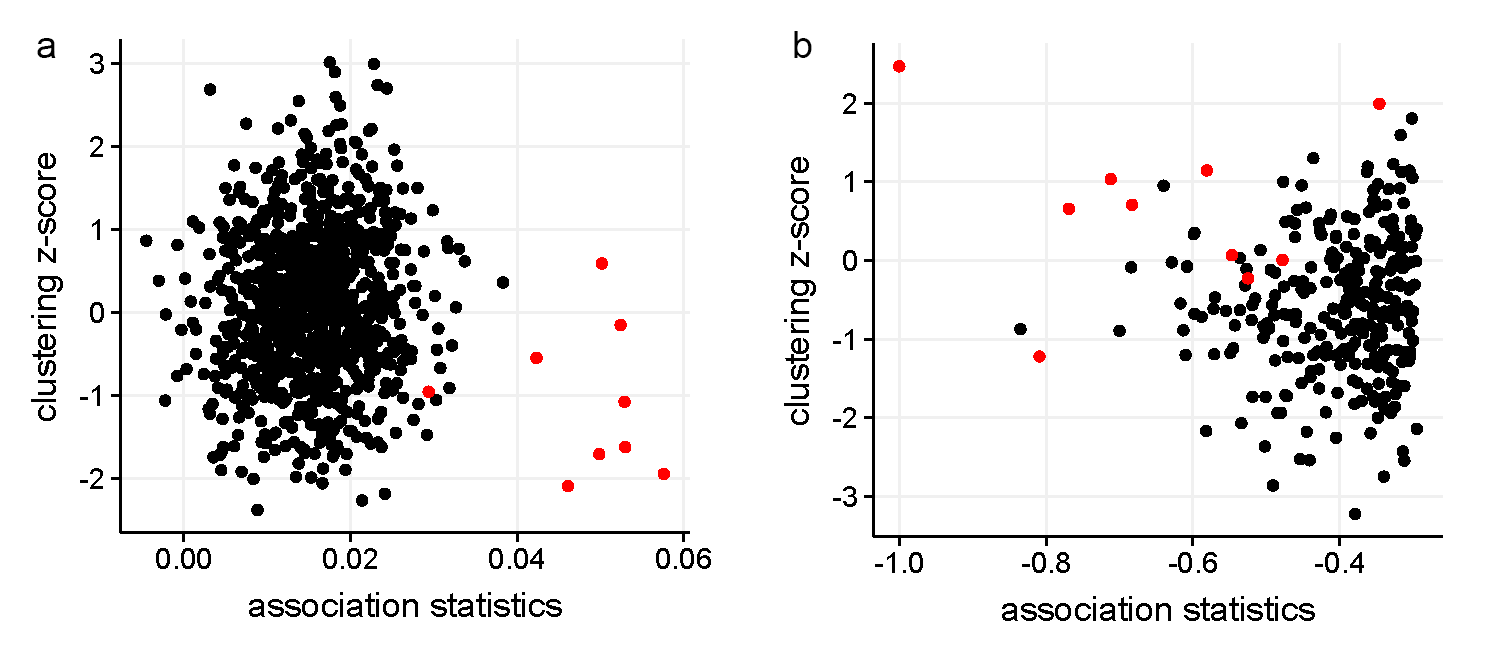

Supplement: S6 Fig — The horizontal axis indicates excess (a) or deficit (b) of rapid consecutive substitutions. The vertical axis indicates the excess of clustering (high positive values of clustering z-score) or repulsion (low negative values of clustering z-score) of non-consecutive substitutions for a site pair. Pairs with positive epistatic interactions have elevated values of the association statistics, and pairs with negative interactions, decreased values. (TIF) [file pgen.1008711.s023.tif]

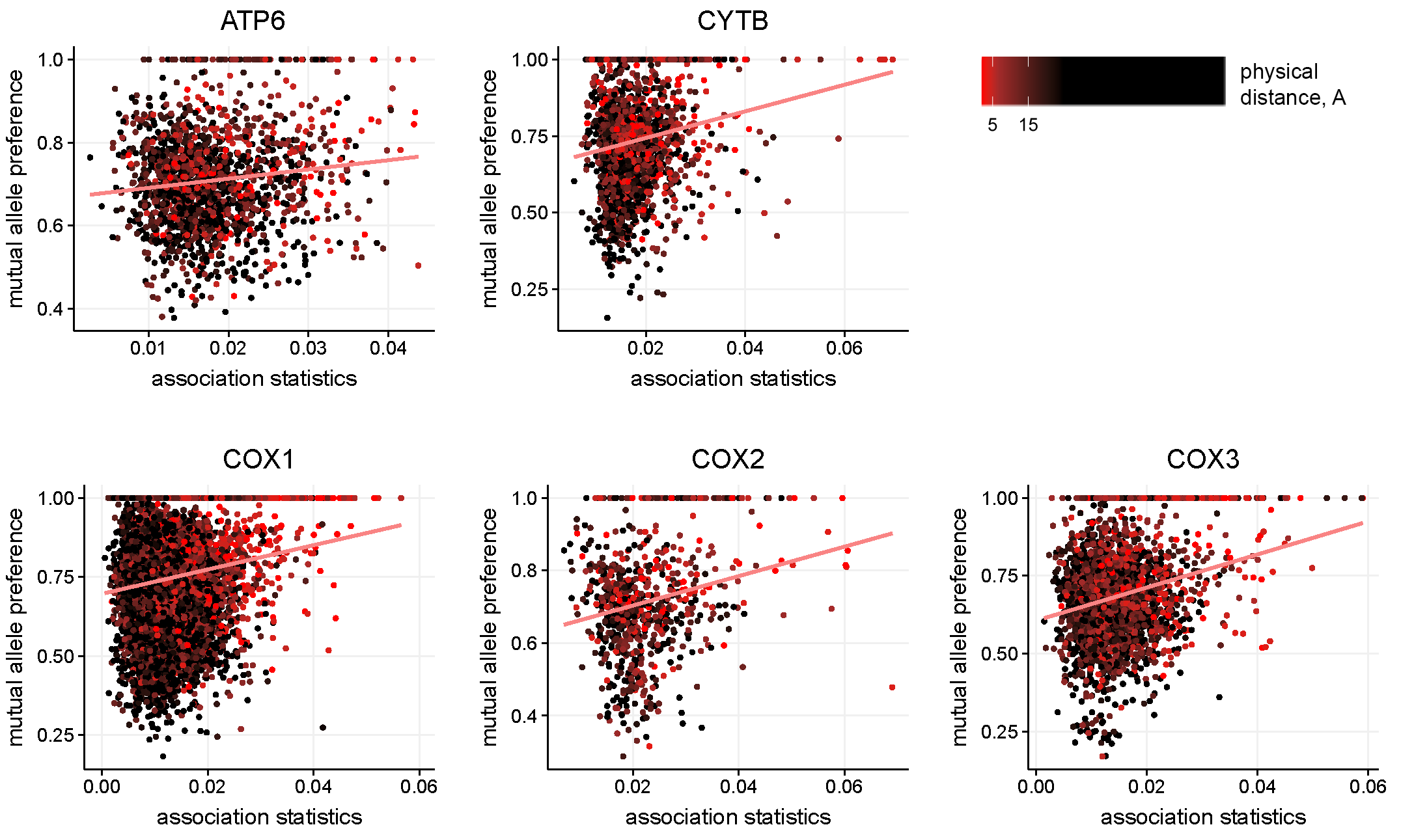

Supplement: S7 Fig — Dot color corresponds to the distance between the sites in 3D protein structures: red for contacting sites and black for distant sites. (TIF) [file pgen.1008711.s024.tif]
