# Supplementary material for: Episodic evolution of coadapted sets of amino acid sites in mitochondrial proteins
Source: PLoS Genet. 2021 Jan 25;17(1):e1008711. doi: 10.1371/journal.pgen.1008711 (PMC7861529; doi:10.1371/journal.pgen.1008711)
Supplement: S1 Data — (ZIP) [file pgen.1008711.s025.zip › data/SI_Data_description.pdf]

Episodic evolution of coadapted sets of amino acid sites in mitochondrial proteins. Supplementary Information: Data description

Alexey D. Neverov [1]*, Anfisa V. Popova[1], Gennady G. Fedonin[1,2,3], Evgeny A. Cheremukhin[4], Galya V. Klink [2] and Georgii A. Bazykin[5,2].

1. Department of Molecular Diagnostics, Central Research Institute for Epidemiology, Moscow, Russia.
2. Institute for Information Transmission Problems (Kharkevich Institute), Russian Academy of Sciences, Moscow, Russia.
3. Moscow Institute of Physics and Technology, Dolgoprudny, Moscow region, Russia.
4. Department of Chemistry, M. V. Lomonosov Moscow State University, Moscow, Russia.
5. Skolkovo Institute of Science and Technology, Skolkovo, Russia.

*The corresponding author: Neverov A.D., mailto: neva_2000@mail.ru

Here we briefly describe contents and formats of files. All data files are partitioned into folders corresponded to content types and to analyses that generated the data. The first-level folders contain raw data (“*alignments*” and “*tree*”) and derived data (“*results*”).

Alignments

The “*alignments*” folder for each gene contains a multiple alignment in the FASTA format of amino acid sequences corresponded to the tree tips and internal tree nodes. The sequences of mitochondria proteins were aligned by Mafft with -einsi option. Sequences of internal nodes were reconstructed by MEGA-CC. The tip nodes are named accordingly to taxonomy Ids of corresponded specimens. The names of internal nodes start from the “Node_” prefix.

Tree

The “*tree*” folder contains the phylogenetic tree in the NEWICK format where names of internal nodes are in the bootstrap slots (“*MEGAtree_mtREV_F.newick*”). The bootstraps are listed separately in the table (“*MegaTreeEinsi.NodeBootstrap*”). For each gene substitutions on the tree branches are provided (see “*.einsi.mega.G10.no_gaps.xparr”).

Substitutions on branches

Each XPARR file contains the phylogenetic tree in the format, where the tree branches are listed in the ancestor-first order. Each row except the first one, which is a root node declaration, contains six columns: a child node id, a parent node id, branch length, a list of synonymous substitutions on the branch – it is empty for protein data, a list of the “;” separated nonsynonymous substitutions and the last column is a copy of the previous one.
Note, that for each alignment of protein sequences only columns which contain less than 10% of internal gaps on the tree tips are presented in XPARR files.

Results

This folder contains subfolders corresponded to results of analyses of distributions of substitutions on the tree: the significant epistatically interacting site pairs (“*site_pairs*”), the partitions of sites into coevolving groups (“*coevolution_graphs*”) and changes of substitution rates in coevolving groups (“*site_groups_tests*”).

Pairs of concordantly and discordantly evolving sites

For each gene significant concordantly and discordantly evolved site pairs are listed in “*.pos.u<upper_pvalue>.tab” and in “*.neg.l<lower_pvalue>.tab” files. The integer parts of p-values (“0.”) in file names are dropped off. The files with concordantly evolved pairs have 7 columns: the first site - “bgr_site”, the second site “fgr_site”, the pseudocorrelation - “element”, the partial correlation - “wi_score”, upper p-value - “upvalue”, lower pvalue - “lpvalue”, the distance between sites on the corresponding pdb structure - “pdb_dist”. The files with discordantly evolved pairs have 6 columns which are the same as listed above with exception of the “wi_score” column which is absent for this type of site pairs. Values of pseudocorrelations are positive for concordantly and negative for discordantly evolved site pairs. For each gene, sites in pairs are numbered according to coordinates in protein alignments. The site pairs in our analysis are unordered, we report coordinates of sites in each pair in increasing order (“bgr_site” < “fgr_site”). Alignment coordinates could be converted into coordinates in the primary sequence of corresponding protein in the PDB structure using the “*.<pdb_id>.align2pdb”. Note, that some alignment sites for some genes couldn't be converted into any site on the structures, it is the cause of empty values in columns “pdb_pos” in some rows of “*.align2pdb” files and in columns “pdb_dist” in “*.tab” files. The PDB identifiers of structures are parts of names of “*.align2pdb” files.

Coevolution graphs

For each gene we provide a coevolution graph in two formats: the PAJEK ("**.net*") and GRAPHML ("**.graphml*"). For the latter format, edges with positive and negative weights are provided in separate files ("**.positive_edges.graphml*" and "**.negative_edges.graphml*"). The PAJEK file could be visualized and analyzed using the Pajek software (http://mrvar.fdv.uni-lj.si/pajek/), the GRAPHML files could be analyzed by igraph (https://igraph.org/).

The subfolder "*partitions*" contains files with partitions of graph vertices into communities by the Modularity method for graphs with signed edge weights ("**.louvain_modularity.out*") implemented in louvain package for Phyton (https://github.com/vtraag/louvain-igraph). We also provide partitions in files suitable for analysis in Pajek ("**.clu*").

The subfolder "*partitions/pdb*" contains script files ("*.rasm") for JMol (**https://sourceforge.net/projects/jmol/files/Jmol/**) visualizing site partitions on protein structures ("*.pdb1"). Sites for each protein have been colored according to their assignments into different coevolving groups.

Tests for substitution rates in coevolving groups

The results of the search of the tree branches that correspond to changes of substitution rates in one or more coevolving groups are presented in the "**.louvain_modularity.groups_test.out*" files. The substitutions rates in groups for each internal tree node are compared to the rates estimated for corresponding closest ancestral node (referred further as current ancestor node) which satisfies to one of two conditions: for that node the significant change of rates has previously been detected or it has been the root node. Each file contains:

- total number of tests which equals to the number of internal tree nodes;
- number of significant tests – the number of branches for which the Bonferroni corrected p-values are below 0.05;
- The search results for each branch of the tree.

For each branch the following information is shown:

- the node id;
- the id of parental node;
- the p-value (uncorrected) of the test;
- the id of the current ancestor node;
- the Kullback-Laibler distance between frequency vectors of substitutions in groups for the node and its current ancestor;
- the id of the group with the greatest change of substitution rates relative the current ancestor (MSG – the shortcut of Max Skewed Group);
- the Kullback-Laibler distance between substitution frequency vector for the node and the model frequency vector. The model assumed that the frequency of substitutions in the MSG group equals to the observed frequency, and frequencies in all other groups are proportional to their frequencies for the current ancestor node. The small value corresponds to better approximation of the group frequencies of the node by the model which assumed that only single group in the frequency vector of the current ancestor node sufficiently changed its frequency and other groups changed their frequencies of substitutions in the same proportion;
- counts of substitutions in groups for the node subtree;

– counts of substitutions in groups for the subtree of the current ancestor node;

– counts of substitutions on the branch corresponded to the node.

Results of application of the test for coordinated changes of substitution rates on the phylogeny for the five mitochondria genes are presented in the file "*summ_site_groups_tests.G10.no_gaps.out*". The "coordinated changes" means that the test for changes of substitution rates has been unexpectedly often rejected for same or sister branches if it has been applied for two or more genes.

The file contains:

– the the number of the parental nodes of the tree branches for which for all of genes the test for changes of substitution rates has been applied;

– for each gene, the number of branches for which the frequency test has been significant

– observed and expected numbers of parental branches for which the frequency test has been significant at least for 0,1,2,3,4,5 genes;

– corresponding p-values;

– The list of parental nodes for which at least for one gene the test for changes of substitution rates in coevolving groups has been significant.

The identifiers of genes in the result file correspond to the ordering numbers of ("**.louvain_modularity.groups_test.out*") files that are listed in the "*groups_test.out.files.tmp*" file.

For each parental node in the result file "*summ_site_groups_tests.G10.no_gaps.out*", the NCBI taxonomy of its daughter nodes is provided in the separate file "*summ_site_groups_tests.G10.no_gaps.node_id2ncbi_taxon.out*", the table consists of

– parental node id;

– the lists of names of taxonomy groups separated by "/", each list corresponds to one of daughter nodes of the parental node; different names of taxonomic categories in each list are separated by "+";

– the full specification of taxonomy category of the parental node.
