# Supplementary material for: Episodic evolution of coadapted sets of amino acid sites in mitochondrial proteins
Source: PLoS Genet. 2021 Jan 25;17(1):e1008711. doi: 10.1371/journal.pgen.1008711 (PMC7861529; doi:10.1371/journal.pgen.1008711)
Supplement: Attachment — Submitted filename: dml.review.docx [file pgen.1008711.s033.docx]

**Summary**

The authors address an important issue in molecular evolution – the distribution of epistasis across sites and the associated properties of epistatic sites. These authors introduce a novel method for detecting negative epistasis in phylogenetic data, which to my knowledge has not been achieved previously. The sign of epistasis is of fundamental importance in evolutionary theory, making this new method an important advance. Interestingly, the authors report that negatively epistatic sites are more common than positive ones and that positively epistatic sites are clustered in proteins structures or involved in inter-protein contacts whereas negatively interacting sites are more distant. However, there are some concerns as to whether the foregoing conclusions can be made safely given potential differences in power of detection and confounding factors such as changing selective forces. Additionally, the interpretation and importance of one of their main results, the coordinated evolution of different proteins, is unclear. Overall, this is solid work but some further analyses to support their claims and substantial editing for clarity are needed.

**Major Comments**

1) The writing, especially in the introduction and the discussion is hard to follow. First, in the introduction, the main purpose of the paper is not clear. Too much background is given, and it is unclear what is most relevant or what issues in the literature they want to address. Second, in the discussion, a concise summary of their main findings and interpretations is not to be found, and their results seem to be lost in the long discussion. The results in the paper are interesting, but much work in the introduction and discussion needs to be done to highlight their importance. It ends up being unclear what the authors want to be their main points.

2) As the authors recognize, phylogenetic methods for detecting epistasis in general suffer from confounders – hitchhiking and more importantly, differing selection pressures across branches or correlated episodes positive/relaxed purifying selection across sites. Previous papers by this group were able to take some steps to estimate the amount hitchhiking might affect their results, by looking at inverse changes, lineage specific positive selection, or corroborating the findings with experimental work. Such steps should be done here, especially as this method is newly applied to negative epistasis.

3) Related to concern in (2), the authors conclude that “negative epistasis appears to be more prevalent than positive epistasis” (line 391). This might be an important point. However, is it possible that their method has more power to detect negative epistasis? Although higher power for negative epistasis would be unexpected, a power comparison should be presented. One issue that might affect power is that positively epistatic sites must occur together within a certain timeframe with a lower bound (simultaneously) and an upper bound (the null rate of co-occurrence). However, for negatively epistatic sites, they must occur more rarely together than expected, and thus only have a lower bound and no upper bound (ie they might never occur together). Might this mean that positive epistasis has to be strong to be found whereas negative epistasis can range from weak to strong? Additionally, might the confounders as in (2) be biased towards negative epistasis for any reason? A discussion or resolution of such issues is needed for the publication of this novel phylogenetic detection of negative epistasis and before making any conclusions about the relative prevalence of the two types of epistasis.

4) The conclusion that negatively epistatic sites are generally more distant than positively epistatic sites seems to rely on Table 3. However, Table 3 does not seem to support such a general conclusion. Only 2/5 genes show a *significant* negative rho for negatively epistatic sites which would support the idea that negative sites are more distant. However, 1/5 genes (Cox1 ) shows a significantly *positive* rho, which supports the opposite conclusion. The remaining two genes show a non-significant negative rho and the other a non-significant positive rho. I don’t see how the general conclusion that negative epistasis is generally associated with higher distances can be made. Of course, all genes support the significant clustering of positive sites. But that does not mean that negatively epistatic sites are more distant than positively epistatic sites. In the absence of clear evidence that negative epistatic sites are significantly not-clustered, a direct statistical comparison between the two types of sites should be made.

The other evidence for negatively epistatic sites being more distant is in the contact graphs showing that contact edges are more numerous within co-evolving (positively epistatic) groups than between groups. This evidence still cannot supplant direct evidence for a difference between negatively and positively epistatic sites, as some pairs of groups may not have any negative interactions.

Perhaps use the network method to group together strong negatively epistatic sites and see if they have more contact edges than random?

5) The purpose of the last set of results “Concordant evolution of groups of sites in different OXPHOS proteins” is unclear. How does this fit in with the epistasis story? The authors state that “this concordance may reflect changes in the selection pressure on the respiratory function” (line 369). Does this actually go against the epistasis story, suggesting that concordant evolution across sties and proteins is due to a correlated change in selection pressures across sites rather than positive epistasis?

**Minor comments**

General editing for English grammar and typos. Also please be more consistent with terms or more clearly define them.. Some examples noted here.

Line 31-32: Generally, *a* newly arisen allele, if it would not be lost, increases its fitness with time due to adaptive mutations occurr*ing* in other sites in the process called ~~the~~ entrenchment

Line 40: “Some of *the blocks* have functional specialization…”

Throughout, it seems that “co-evolving” sites or groups refers to positively epistatic sites. Please define this “co-evolving” usage somewhere or stick to “positively epistatic” sites/groups.

*Author Summary*

Line 32: A specific example of what I mean by the unfocused writing (major comment 1): here entrenchment is mentioned but in the main text the entrenchment concept is never discussed again.

*Intro*

Line 64: “rigid dynamics domains”?

Line 72: What is it meant by “how these associations manifest themselves during course of evolution?”

*Results*

Figures 1 and 2 don’t help to show their method. For example, figure 1 shows red and blue branches, substitutions occurring more or less often than expected…but figure 1 is supposed to show concordant positive epistasis signals. And the difference between figure 1 and 2 is not clear, especially why one is supposed to show positive vs negative (concordant vs discordant) signals. Additionally, are the dots representing the substitutions at the sites in question? Hard to understand.

Perhaps as a different figure 1/2, a figure explaining the difference between this method and others that allowed this paper to uniquely detect negative epistasis would be very helpful and drive home the novelty of the paper. This would also make their depiction of their current method clearer.

Figure 4 – the authors state that residues involved in inter-protein interactions are these spheres, those not involved in interprotein interactions are ribbons, and the “remaining” in grey. I presume those “remaining” are sites not on the surface. Why not simply state “and internal sites not involved in interprotein interactions are in grey.”

Figure 4 – The point of the bar graphs is to see if there is a difference between expected and observed. However, the presentation of the data make it hard to get the general trend from the plot and see if the data support their conclusion. Please plot the difference between observed and expected. The raw numbers can be plotted in a supplemental figure.

Line 219 – Define “density” of edges

*Discussion*

Why were previous papers unable to detect negative epistasis, with what seems to me a very similar method? What, exactly, was different about this method? Or was it the data that made the crucial difference? Please discuss; it pertains to the applicability of the method for other genes/phyla.

Can you speculate on why negative epistasis may be associated with more distant sites versus positive epistasis? Does this relationship with distance explain the asserted greater prevalence of negative epistasis – eg, given the protein structures, are the more pairs of distant sites than pairs of clustered sites?

Lines 354 – 374: Confusing paragraph

Line 376: Use consistent terms, in this case negative rather than “synergistic epistasis.

Line 376: Discussion of “seeding” mutations and constraining the number of evolutionary pathways. Must be referring to sign epistasis? However, not sure if discussion pertains to the findings. The negative epistasis found is not necessarily sign epistasis, a mutation may just diminish the adaptive benefit of the other mutation rather than change its sign. Can the authors detect stronger negative epistasis, actual sign epistasis? Would be quite interesting if so.
