# Supplementary material for: Episodic evolution of coadapted sets of amino acid sites in mitochondrial proteins
Source: PLoS Genet. 2021 Jan 25;17(1):e1008711. doi: 10.1371/journal.pgen.1008711 (PMC7861529; doi:10.1371/journal.pgen.1008711)
Supplement: Attachment — Submitted filename: responce_to_rewiers.docx [file pgen.1008711.s034.docx]

### Reviewer #1:

In this manuscript, the authors modified an algorithm from a previous study, to infer pairs of amino acid sites that experienced excessive or deficient amount of correlated substitutions, along phylogenetic trees for mitochondrial proteins. They further found that concordant sites are spatially close to each other while discordant sites are far away on protein structures. The authors then clustered sites into coevolving groups and indicated that some groups are related to protein surface interaction, and the burst of substitutions for groups happen at some branches more than the others among different OXPHOS proteins.

The pattern and mechanism of site coevolution in protein have been of wide interest. While this study conducted detailed analyses, I recommend the authors discuss more about the comparison between existing studies and their findings, to reflect the novelty of their work. For example, Levin and Mishmar published a study in 2017 on Nature Ecology and Evolution also on OXPHOS proteins and other genes, discovering extensive compensatory substitutions happening on related branches of large phylogeny, affecting amino acid sites within a close physical distance with each other in 3D structure. This should be cited and discussed.

**Thank you for pointing us to this important reference; we now cite this work in the Introduction.**

Below are detailed comments.

Line 25. “mitochondrially encoded” should be “mitochondrion-encoded”.

**Corrected.**

Line 157. The authors claim that “At most of the negatively associated site pairs, the substitutions at the two considered sites tended to occur in lineages that were not only distinct, but also remote from each other on the phylogeny”. Is it supported by statistical test, i.e. such substitutions are more distant on the tree than expected?

**We now remove this statement, and instead add a new Results section (“Concordant and discordant evolution can be caused by episodes of epistatic or additive selection”) analyzing the mutual position of non-consecutive substitutions.**

Line 159. For both table 1 and table 2, instead of a table showing number of sites at different P-value cutoff, a histogram showing the distribution of P-values of all sites might provide more information on the false discovery.

**As suggested, we now present these data as figures (Supplementary Figs. S1-S2).**

Also, there should be multiple testing correction for FER.

**The false exceedance rate (FER) is by itself a method of control for multiple testing (an alternative to the FDR); see refs. 84, 85 . Indeed, the FER is a tail probability of FDR, i.e. FER=P(V/R > q), where V/R is the fraction of false positives (V) among the declared positives (R), and 0<=q<=1 is a parameter that controls the stringency of the procedure. We set q=1; that means that we estimate the probability to make more discoveries among the datasets sampled from the null model than in the experimental data P(V>R). If the FER>0.05 for some nominal p-value threshold, we conclude that there is no signal in the experimental data at this threshold value. Thus, we don’t need to additionally control for multiple testing for FER.**

Line 211. Is the “spurious” correlation controlled here referring to the analysis at Line 443? If yes, this might need to be clarified in the main text; if not, what is the criteria for spurious here?

**No, this is a different problem; now clarified. At line 211, we discuss a correlation between two sites that occurred due to these sites being strongly correlated with a third site. At line 443, we talked about substitutions on tree branches that were artifacts of uncertainty of phylogenetic reconstruction.**

Line 224. In Table 5, the group 4 of atp6 contain only 1 site. Is this a valid coevolving group?

**This is a formal result: the vertex clustering algorithm allows groups containing only a single vertex, and this is what happened here. Biologically, this implies that this site evolves discordantly from sites in other groups.**

Table 5 has no multiple testing correction for the P-value despite large number of statistical tests.

**We now applied the Benjamini–Hochberg correction at the 5% alpha level for tests for individual groups. The p-value threshold corresponding to this correction was 0.042; this is now mentioned in the caption to Table 5. We also applied the (more conservative) Bonferroni correction to the p-values obtained for individual genes. The tests passing the mutliple testing correction are now identified.**

Also, the authors didn’t mention by what test the p-values were derived.

**The P-values for groups of sites to be enriched by contacts in the protein structure are obtained by permutation test. The procedure is explained in the section “Overlaying coevolution and contact graphs” of the Methods. Briefly, we construct a contact graph where vertices are sites and edges are contacts on the structure. The group statistic is the fraction of edges where both sites belong to the same group, among the edges that have at least one site in the group. The statistics for the entire protein is defined similarly, as the fraction of edges of a contact graph having both edges within one group among all edges. To calculate the p-values, we compare the statistics obtained for the data to the distribution of statistics obtained from permutations in which each site is randomly assigned to one of the groups.**

Line 243. For P<0.05 threshold used here, no multiple testing correction is conducted.

**We now applied the multiple testing corrections as detailed above, and indicated in the Table 5.**

Line 256. “APT6” should be “ATP6”

**Done.**

Line 280 “where” should be “were”

**Done.**

Line 435. “in a random dataset”: how is this random dataset constructed?

**These are the same 400 random sets discussed above; now clarified.**

Line 507. “chi^2” should be “chi square”

**Done.**

In Figure legends, panel index (a, b, c, etc) are inconsistent with those in the Figure (A/B/C…)

**Corrected**

Line 851. second “left” might be “right”

**The figure legend is correct as stated. The contact graph is schematically represented in the left, while the coevolution graph is represented in the middle (edges with positive weights) and in the right (edges with negative weights). We changed the title of the figure 4 to emphasize the order of graphs shown on the figure: “Schematic representation of contact and coevolution graphs for COX1 (a-c), COX2 (d-f), COX3 (g-i), ATP6 (j-l) and CYTB (m-o) proteins”.**

Line 861. Figure 4 does not have panel d, e, f.

**Corrected**

Line 870. “… function of ATP synthase” should be “… function of ATP6 in ATP synthase”

**Corrected.**

**==================**

### Reviewer 2

**Summary**

The authors address an important issue in molecular evolution – the distribution of epistasis across sites and the associated properties of epistatic sites. These authors introduce a novel method for detecting negative epistasis in phylogenetic data, which to my knowledge has not been achieved previously. The sign of epistasis is of fundamental importance in evolutionary theory, making this new method an important advance. Interestingly, the authors report that negatively epistatic sites are more common than positive ones and that positively epistatic sites are clustered in proteins structures or involved in inter-protein contacts whereas negatively interacting sites are more distant. However, there are some concerns as to whether the foregoing conclusions can be made safely given potential differences in power of detection and confounding factors such as changing selective forces. Additionally, the interpretation and importance of one of their main results, the coordinated evolution of different proteins, is unclear. Overall, this is solid work but some further analyses to support their claims and substantial editing for clarity are needed.

**Major Comments**

1) The writing, especially in the introduction and the discussion is hard to follow. First, in the introduction, the main purpose of the paper is not clear. Too much background is given, and it is unclear what is most relevant or what issues in the literature they want to address. Second, in the discussion, a concise summary of their main findings and interpretations is not to be found, and their results seem to be lost in the long discussion. The results in the paper are interesting, but much work in the introduction and discussion needs to be done to highlight their importance. It ends up being unclear what the authors want to be their main points.

**We revised the text for clarity, streamlining the introduction and clarifying our focus in the introduction and discussion.**

2) As the authors recognize, phylogenetic methods for detecting epistasis in general suffer from confounders – hitchhiking and more importantly, differing selection pressures across branches or correlated episodes positive/relaxed purifying selection across sites. Previous papers by this group were able to take some steps to estimate the amount hitchhiking might affect their results, by looking at inverse changes, lineage specific positive selection, or corroborating the findings with experimental work. Such steps should be done here, especially as this method is newly applied to negative epistasis.

**In response to this criticism, we have now added extensive new analyses asking whether the observed concordance or discordance could result from episodic selection (uncorrelated between sites), clonal interference, or hitchhiking. Specifically, we now add a new section in the Results detailing three types of simulations, using two different methods, which indicate that these phenomena by themselves do not lead to a signal of concordance or discordance. Notably, episodes of selection correlated between sites may result both in a pattern of concordant and discordant evolution; we take additional steps to distinguish the two, adding another section to the Results on that (see below).**

**As a side note, all these mechanisms could conceivably lead to concordance between sites, but even theoretically it is unclear why they would lead to systematic discordance between sites (negative epistasis), which is the main focus of this paper. This is now discussed.**

3) Related to concern in (2), the authors conclude that “negative epistasis appears to be more prevalent than positive epistasis” (line 391). This might be an important point. However, is it possible that their method has more power to detect negative epistasis? Although higher power for negative epistasis would be unexpected, a power comparison should be presented. One issue that might affect power is that positively epistatic sites must occur together within a certain timeframe with a lower bound (simultaneously) and an upper bound (the null rate of co-occurrence). However, for negatively epistatic sites, they must occur more rarely together than expected, and thus only have a lower bound and no upper bound (ie they might never occur together). Might this mean that positive epistasis has to be strong to be found whereas negative epistasis can range from weak to strong? Additionally, might the confounders as in (2) be biased towards negative epistasis for any reason? A discussion or resolution of such issues is needed for the publication of this novel phylogenetic detection of negative epistasis and before making any conclusions about the relative prevalence of the two types of epistasis.

**Thank you for this important point. After considering it carefully, we decided to withdraw our statement about the relative prevalence of positive and negative epistasis in Results and Discussion. Indeed, we are unable to compare the prevalence of the two classes of epistasis without comparing the abilities of our test to detect each class of epistatic interaction. To do that, we would need to model the evolution of mitochondrial proteins with two classes of epistasis between sites directly. Simulating epistasis is notoriously difficult, and any such simulation will be very sensitive to the assumptions made. To our knowledge, there is no appropriate simulation framework for this task.**

4) The conclusion that negatively epistatic sites are generally more distant than positively epistatic sites seems to rely on Table 3. However, Table 3 does not seem to support such a general conclusion. Only 2/5 genes show a *significant* negative rho for negatively epistatic sites which would support the idea that negative sites are more distant. However, 1/5 genes (Cox1 ) shows a significantly *positive* rho, which supports the opposite conclusion. The remaining two genes show a non-significant negative rho and the other a non-significant positive rho. I don’t see how the general conclusion that negative epistasis is generally associated with higher distances can be made. Of course, all genes support the significant clustering of positive sites. But that does not mean that negatively epistatic sites are more distant than positively epistatic sites. In the absence of clear evidence that negative epistatic sites are significantly not-clustered, a direct statistical comparison between the two types of sites should be made.

**Thank you for this point. As suggested, we now make a direct comparison between significantly concordantly and discordantly evolving site pairs. For all genes, the mean distances for concordant pairs are significantly lower than those for discordant ones (Mann Whitney U-test, P<2e-16; tab. S3). This is now discussed. The numbers of contacting pairs for site categories are now also detailed in Table 3.**

The other evidence for negatively epistatic sites being more distant is in the contact graphs showing that contact edges are more numerous within co-evolving (positively epistatic) groups than between groups. This evidence still cannot supplant direct evidence for a difference between negatively and positively epistatic sites, as some pairs of groups may not have any negative interactions.

Perhaps use the network method to group together strong negatively epistatic sites and see if they have more contact edges than random?

**Thank you for this suggestion. As suggested, we now design a procedure to group together strong negatively epistatic sites. For this, we inverted the signs of edge weights for the coevolutionary graphs and applied the same vertex clustering procedure which has been applied to the original graphs. None of the genes has significant (p<0.05) enrichment of identified groups of sites by contacting site pairs. A similar test for individual groups has a relatively low p-value (0.03) only for one out of the 26 groups, and even this result does not survive the multiple testing correction. Therefore, we conclude that, unlike positively epistatic pairs, the negatively epistatic pairs do not have an excess of contacts.**

5) The purpose of the last set of results “Concordant evolution of groups of sites in different OXPHOS proteins” is unclear. How does this fit in with the epistasis story? The authors state that “this concordance may reflect changes in the selection pressure on the respiratory function” (line 369). Does this actually go against the epistasis story, suggesting that concordant evolution across sites and proteins is due to a correlated change in selection pressures across sites rather than positive epistasis?

**In the revision, we significantly revise the manuscript to account for the possibility that our results are partially driven by non-epistatic episodic evolution. Specifically, we add a new Results section focused on disentangling the epistatic and non-epistatic episodic evolution, and show that non-epistatic episodes of selection contribute to our results. This makes the link between the last section of the Results and the rest of the paper clearer. We also introduce multiple textual changes to reflect this.**

**Minor comments**

General editing for English grammar and typos. Also please be more consistent with terms or more clearly define them.. Some examples noted here.

**We revised the text, hopefully improving it.**

Line 31-32: Generally, *a* newly arisen allele, if it would not be lost, increases its fitness with time due to adaptive mutations occurr*ing* in other sites in the process called ~~the~~ entrenchment

**Corrected**.

Line 40: “Some of *the blocks* have functional specialization…”

**Corrected**.

Throughout, it seems that “co-evolving” sites or groups refers to positively epistatic sites. Please define this “co-evolving” usage somewhere or stick to “positively epistatic” sites/groups.

**Now we add paragraphs into the Results and Discussion sections where we explain that the signal of concordant and discordant evolution in our data could be caused by concurrent episodic selection and epistatic interactions. We provide evidence that the episodic selection alone could not explain all of our findings and epistasis certainly takes place. Now we used the term of “coevolving” groups of sites only in the context of partitioning of sites into groups such that sites within groups are mainly concordantly evolved and sites in different groups are evolved discordantly.**

*Author Summary*

Line 32: A specific example of what I mean by the unfocused writing (major comment 1): here entrenchment is mentioned but in the main text the entrenchment concept is never discussed again.

**We now no longer mention entrenchment.**

*Intro*

Line 64: “rigid dynamics domains”?

**Changed to “sites in these domains have cooperative dynamics”.**

Line 72: What is it meant by “how these associations manifest themselves during course of evolution?”

**We have thoroughly changed this paragraph. This phase is absent in the revised text.**

*Results*

Figures 1 and 2 don’t help to show their method. For example, figure 1 shows red and blue branches, substitutions occurring more or less often than expected…but figure 1 is supposed to show concordant positive epistasis signals. And the difference between figure 1 and 2 is not clear, especially why one is supposed to show positive vs negative (concordant vs discordant) signals. Additionally, are the dots representing the substitutions at the sites in question? Hard to understand.

**The figures 1 and 2 in the original manuscript depict examples of concordantly and discordantly evolved site pairs. In response to suggestions we add two schematic figures referred to as figure 1 and figure 2 in the revised version illustrating the main ideas of our analysis. The examples of concordantly and discordantly evolving site pairs now are shown on figures 4 and 5. On each of these figures we add a legend explaining the meaning of dot colors. Each figure has a detailed description. As suggested by journal Submission Guidelines, the captions of figures are grouped together in the separate section before the figures itselves.**

Perhaps as a different figure 1/2, a figure explaining the difference between this method and others that allowed this paper to uniquely detect negative epistasis would be very helpful and drive home the novelty of the paper. This would also make their depiction of their current method clearer.

**See figures 1 and 2 in the revised version of the manuscript.**

Figure 4 – the authors state that residues involved in inter-protein interactions are these spheres, those not involved in interprotein interactions are ribbons, and the “remaining” in grey. I presume those “remaining” are sites not on the surface. Why not simply state “and internal sites not involved in interprotein interactions are in grey.”

**The “remaining” sites are either internal sites or those that were not analyzed, e.g. because they had no significantly concordantly evolved counterparts. We now clarified that in the figure legend.**

Figure 4 – The point of the bar graphs is to see if there is a difference between expected and observed. However, the presentation of the data make it hard to get the general trend from the plot and see if the data support their conclusion. Please plot the difference between observed and expected. The raw numbers can be plotted in a supplemental figure.

**Thank you for the suggestion, but we would like to leave the figure unchanged, because the height of bars reflect the sizes of site groups that, as we hope, helps to juxtapose bar charts with protein structures.**

Line 219 – Define “density” of edges

**We now add the definition of edge density into this paragraph: “Here, within group edge density was defined as the ratio of total weight of graph edges connecting vertices from the same group to the total weight of all edges, and the inter-group edge density was defined as the complementary value.”**

*Discussion*

Why were previous papers unable to detect negative epistasis, with what seems to me a very similar method? What, exactly, was different about this method? Or was it the data that made the crucial difference? Please discuss; it pertains to the applicability of the method for other genes/phyla.

**To the best of our knowledge, no attempt has been made previously to tell the mechanism underlying the observed coevolution patterns in mitochondria proteins, e.g. to tell negative from positive epistasis. The exception is ref. 36 now discussed in the Introduction.**

**In our own previous work based on phylogenies, our focus was on positive epistasis, and we did not attempt to extract the signal of “repulsion” of consecutive substitutions from each other. The question asked here is new. The method is applicable to any phylogenies with sufficient data, and further work is necessary to understand the prevalence of negative epistasis in other systems.**

Can you speculate on why negative epistasis may be associated with more distant sites versus positive epistasis? Does this relationship with distance explain the asserted greater prevalence of negative epistasis – eg, given the protein structures, are the more pairs of distant sites than pairs of clustered sites?

**In response to your criticism, that without direct comparison of powers of the method to detect concordantly and discordantly evolved site pairs, we could not surely talk about prevalence of negative epistasis on the positive one, we now withdraw our statement.**

Lines 354 – 374: Confusing paragraph

**We revised the discussion and we hope that now this paragraph is in the appropriate context.**

Line 376: Use consistent terms, in this case negative rather than “synergistic epistasis.

**Indeed, synergistic epistasis between deleterious mutations is negative epistasis; removed “synergistic” as suggested.**

Line 376: Discussion of “seeding” mutations and constraining the number of evolutionary pathways. Must be referring to sign epistasis? However, not sure if discussion pertains to the findings. The negative epistasis found is not necessarily sign epistasis, a mutation may just diminish the adaptive benefit of the other mutation rather than change its sign. Can the authors detect stronger negative epistasis, actual sign epistasis? Would be quite interesting if so.

**As we cannot measure the selection coefficient associated with each mutation using our method, we are unfortunately unable to tell whether the observed negative epistasis is sign or magnitude epistasis. Our discussion of seeding mutations provides an example of a reason why negative epistasis could favor longer-distance interactions, compared to positive epistasis; but we do not suggest that this is the actual mechanism. Furthermore, for this mechanism to operate, sign epistasis is not required - strong magnitude epistasis should suffice (e.g., if one of the two seeding mutations renders the other one useless).**

### Reviewer #3:

This work builds on previous studies from this group (refs 36 and 37) in detecting epistasis based on the timing of mutational events. The authors include this approach to detect positive interactions, and further extend these analyses to detect negative epistatic interactions.

An important feature of this paper's approach is that instead of using time-resolved evolution of influenza sequences, they use 5 OXPHOS pathway proteins. OXPHOS is a classic model where phylogenetic, structural and cellular biology can be used to understand epistasis and co-evolution. Thus another defining feature of this study is that while the earlier influenza model evolved over a microevolutionary timescale of ~ 30 years, OXPHOS evolution occurred over a macroevolutionary timescale.

While I find the new approach and model intriguing, it was difficult for me to follow exactly what analyses the authors performed, in particular how the statistics are calculated, especially this new negative epistatic statistic. Part of this is because the authors simply refer back to their earlier publications for methodological and even broader ideas, expecting the reader to also read those papers in depth.

**Thank you for the precise review of our manuscript. We now took effort to clarify our approach, particularly by including two schematic figures illustrating it (Figs. 1 and 2 in the revised manuscript). We also revised the description of the problem and methodology, hopefully making the manuscript self-contained.**

Thus the impact of this paper depends heavily upon the strengths and frailties of this group's earlier publications, a kind of "literature epistasis" which could be avoided by being more direct and explicit in describing their methods and broader context of the current study.

As a result I unfortunately cannot really comment on the quality of the analysis or conclusions of the study, especially the new model of detecting negative epistasis. I would recommend a heavy rewrite clarifying methodology, including better qualitative descriptions of what was done, quantitative descriptions (equations), more clear null models, and the sharing of code, at least for calculation of their "statistics".

**We now add Supplementary Methods formally describing the procedure without the need to refer to our previous papers. Also, we deposited the code on Github and provided files that are required to reproduce our analyses (**<https://github.com/gFedonin/EpiStat>)**.**

I also think that at the core of this methodology is an assumption that the timing of mutational events reflects epistasis, when it might simply reflect a coevolutionary processes which could include additive or epistatic interactions. Orthogonal analyses, including generative null models which include different scenarios of epistasis, might more convincingly support their conclusions that the observed phenomenon is epistasis per se.

**In the revision, we include new analyses and discussion aimed at disentangling additive and epistatic episodic selection; see response to Reviewer 2 (point 5) and details below. We also include new simulations to show that the observed clustering is not explainable by stationary evolution; see response to Reviewer 2, point 2.**

Finally, I think the paper would benefit throughout, in figures and text, from clarification of the major discoveries made using this new approach and model system, including clarification of the "LEGO" analogy, discussion of what new insights negative epistasis provided, and the power of using phylogeny in large protein families which span long evolutionary distances.

**Thank you for suggestions. We thoroughly revised our manuscript for clarity. We also decided to remove “LEGO” from the Authors Summary because this analogy didn’t include in itself more than presence of structural organization of protein sites into domains or “sectors” (these terms are often used in the literature).**
------------------------------------
Major comments
------------------------------------

1) Please clarify methods and share code.

The methods section requires an extensive rewrite to a far greater depth than it is at present.

**We now revised methods for clarity, added a new Supplementary Methods section, and deposited the code to Github (**<https://github.com/gFedonin/EpiStat>)**.**

Two major points:

A) In the text there are two usages of the word "statistic": an "association statistic" and an "epistatic statistic". Neither of these are described quantitatively (or clearly qualitatively, really) anywhere in the text. It is not OK to ask the reader to read reference 36 and guess where its methods apply here.

**Thank you for this comment. We now added the Supplementary Methods section, where we describe formally the methodology of analyses and statistics. The manuscript no longer requires the reader to refer to our previous papers.**

This problem snowballs later on, for example in interpretation of their "pseudo-correlation" which is the "sum of epistatic statistics for a site" normalized by the max if positive and min if negative (??), which seems unconventional to me given the potential for massive scale variation between sites.

I gave up here because I couldn't really follow the most basic analyses they performed.

**We clarify our statistics in Supplementary Methods. The normalization is simply by the maximum absolute value; now revised for clarity.**

B) In the text the authors mention a "null" however I cannot find a description of how this was generated. The earlier work from this group benefitted from an extensive methods section and null distribution produced by a generative model as well as a data permutation approach. Do establish that they are detecting epistasis, and not simply signals of coevolution, I believe that these null models should include specific models of epistasis, and whether their signatures should be detectable in simulated datasets.

**In the revision, we now include three additional generative null models showing that our signal does not arise spuriously from hitchhiking, clonal interference, or episodic selection that is uncorrelated across sites (see response to Reviewer 2, point 2). Modelling epistasis in a phylogenetic framework is notoriously difficult and computationally intensive; such a model will also be very dependent on the details of parameter choice, and merits an independent study. Our main argument is that the observed patterns signify epistasis, and therefore using non-epistatic null models seems appropriate.**

I mention other points at the bottom of the text here in the "Methods comments" section.

Finally, it's great the authors included the data, especially the alignments and trees, but in the case of a computational study like this, where much of the data were generated by computer programs, the code used to generate the data is critical to interpret and replicate the work. It is increasingly unacceptable to publish something like this today without sharing the code used to support the conclusions.

**We now deposit the code to Github and provide files required to reproduce our analyses (**<https://github.com/gFedonin/EpiStat>)**.**

2) Validity of the 'inferring epistasis via timing' approach.

I feel the authors should indicate whether they can detect epistasis per se, and include an analysis of whether different types of epistatic interactions can be detected using their approach.

First point - Is it epistasis?

I believe the authors should more carefully consider the assumption that mutations which co-occur (or do not co-occur) in time happen due to epistasis. This is because co-evolution does not require epistasis. Epistasis means that the _effect_ of a mutation depends upon other genetic sequences. So, one could have co-evolving sites which additively sum to make a fit phenotype and thus do not have any dependency in their effects (see for example additive coevolution at linked sites recently from Rockman <https://onlinelibrary.wiley.com/doi/full/10.1002/evl3.139>).

**Thank you for this insightful comment. We agree that co-occurrence of substitutions may result both from epistasis and non-epistatic (additive) episodic evolution, and now discuss this distinction in detail. In the revision, we add a Results section aimed to discriminate between these factors.**

**First, we note that epistatic and non-epistatic episodic evolution can be distinguished based on the phylogenetic patterns they cause. For example, positive epistasis is only expected to lead to phylogenetic “clustering” for pairs of consecutive substitutions, and is not expected to bias the phylogenetic distribution of substitutions that do not fall into the same lineage. By contrast, episodic selection, e.g. acting within a certain clade, is expected to bias the substitution patterns at all sites affected by this selection within this entire clade, independent of whether these substitutions occur in the same or in different lineages.**

**We now design a framework to distinguish between these scenarios. Overall, we see that the patterns observed at concordant and discordant site pairs differ between genes. For some genes, they indicate non-epistatic episodic evolution, while for others, epistasis has to be invoked. We now introduce changes throughout the manuscript to reflect the fact that both these factors operate.**

**We also provide several additional lines of evidence that epistasis actually does contribute to our results. First, as already discussed in the original submission, the spatial proximity between sites of substitutions in protein structures is more consistent with the epistatic explanation.**

**Second, in the revision, we now add additional analyses to show this. For pairs of sites where substitutions co-occur, we show that the substitutions at the second (“trailing”) site are such that the resulting amino acid is statistically unlikely, i.e., substitutions into this amino acid at this site are rare. Importantly, at all five considered proteins, this effect is the strongest for pairs of contacting sites, again supporting the epistatic explanation for our observations.**

Thus, simply because two sequences are observed to change together in a coordinated fashion does not mean that one of the mutations' effects depended on or was altered by the other - they could combine in an additive manner. Similarly, just because two deleterious mutations are seen to not combine together does not mean that their combined product is worse than one would expect alone - it's just that two bad mutations are additively bad. Thus it is possible that the authors' "epistasis" scores simply detect coevolution alone.

**Here, we have to disagree: two bad mutations that are additively bad will NOT be seen to not combine together any more than randomly expected. This is because phylogenetic biases are caused by changes in substitution probability, and this in turn is determined by the selection coefficient, which, in the absence of epistasis, is by definition independent of the genetic background. Therefore, a phylogenetic bias in the distribution of substitutions in a pair has to imply changes in selection - either caused by epistasis or not.**

Unless I am missing an important detail in these analyses, I think this remains an outstanding problem with the authors' approach. If I understand the generative "Simulations without epistasis" null model presented in reference 36, different types of genetic interactions (additive, negative, positive, suppressing interactions) were not explicitly modeled, and therefore we do not know if we can distinguish epistasis from simple co-evolutionary signal that might include additive and epistatic effects.

**Indeed, we do not explicitly model epistasis; our null model is limited to additive (non-epistatic) interactions. We now provide arguments that both epistasis and non-epistatic episodic selection contribute to our results (see above).**

Second point - "Negative epistasis"

As the authors point out in their final paragraph (line 395) "negative epistasis has, to our knowledge, not been reported from similar data, probably because detection of a deficit of events is harder than detection of an excess"

I cannot comment on the negative epistasis score they present here because its calculation was not described. However, drift and demography seem like the most likely reasons that one would get spurious signals of mutual exclusivity. I hope in the future that the authors address this directly in the text and with figures and code.

**We now clarify the description of the negative epistasis score (see Supplementary Methods). We also include additional generative null models for non-epistatic evolution, showing that they cause no signal of epistasis.**

**Regarding the effect of drift and demography, they are not expected to lead to a non-uniform substitution rate. Indeed, according to the Kimura’s basic theorem of neutral evolution, the neutral substitution rate equals the mutation rate, and is independent of demography (or anything else) (Kimura 1983). Perhaps what the Reviewer means is weakly deleterious substitutions; their rate can indeed depend on the population size, with more substitutions in smaller populations (Ohta 1970). There is no way, however, this can lead to the “repulsion” signal of negative epistasis that we see.**

In sum, I think the paper, and growing body of work presented by this group in relation to how the timing of mutational events informs about coevolution, would benefit from a deeper discussion of different quantitative genetic and evolutionary scenarios where this method is strong and could fail. Simulation of the evolutionary process under different models of the effects of mutation combinations might be required to address this problem. For these, not only the branching process but also the type of genetic interaction (additive, negative, positive, suppressing interactions) would benefit the analyses.


------------------------------------
Minor comments
------------------------------------

- How do the authors independently support their ability to detect negative epistatic interactions? For example, in the case of negative epistasis between a and b positions, when deleterious mutation a -> A occurs, the sequence at another position b becomes more essential than it was before because it is less tolerant to other b -> B mutations. If this were the case, should it lead to a signal of purifying selection at b (and linked/coevolving sites?) in 'A' lineages compared to 'a' lineages? Or am I describing the analysis itself here?

**Exactly – this is the idea of our analysis. We hope this is clearer in the revision.**

- Why leave out leading and trailing sites in the analysis (line 422)? The "directional epistasis" described earlier by this group was impressive because it implied a true epistatic interaction. I don't think the authors have formalized the argument, but a two-locus suppressing relationship, where mutation at locus a -> A is deleterious, but rescued by otherwise neutral b -> B mutation, would lead to ab -> aB -> AB genotype orders but not a -> aB -> AB in phylogenies. (other explanations could be either changing background mutations which alter B's effect, drift, or environmental change).

**We pooled together reciprocal site pairs (a->A followed by b->B and b->B followed by a->A) as this allowed for more statistical power. The ordered site pairs consisting of leading and trailing sites also yielded a signal, albeit a weaker one. This might suggest that much of the epistasis we observe is reciprocal. However, there are confounders; for example, many of the observed pairs of substitutions occur at the same branch (see sand-colored dots on the trees on fig. 4 and 5 ), so we do not know which came first (see also next comment); the statistics for unordered pairs may work better for those. We believe that it is difficult to infer the detailed properties of the epistatic landscape (besides it being positive or negative) from our data, and prefer not to make any claims about them.**

Apart from drift, for influenza, the suppressing 2-locus landscape or changing environment hypotheses seem most likely, because it seems unlikely that the genetic environment could change so dramatically over periods of < 30 years. However, over hundreds of thousands to millions of years as in the OXPHOS dataset, perhaps other scenarios are more likely?

**In the revision, we now discuss in detail the alternative explanation for our results - non-epistatic episodic evolution - and argue that it cannot explain our observations fully, leaving some role for epistasis. Regarding the timescale, we note that most of the signal for concordant evolution came from substitution pairs that occurred on the same branch. For all we know, these substitutions could occur very rapidly after one another. Also, we note that the mutation rates are very different between the influenza and mitochondrial genomes, and the difference in accumulated divergence is lower than that in timespan.**

- For macroevolutionary timescales such as the OXPHOS system, detecting epistasis by "timing" might be prone to problematic inferences due to a process similar to incomplete lineage sorting, whereby rapidly evolving (highly diverse) ancestral sequences might lead to sequences in divergent branches which appeared to independently evolve when in fact these sequences were present at low frequencies in ancestral populations prior to divergence. Some of the authors have previously worked with a similar issue in studying homoplasy. Was this a likely problem in the current study, and did the authors address it directly?

**The issue of hemiplasy is addressed by our analysis - see the first section of the Results and Table S1. Of note, the actual incomplete lineage sorting is unlikely for most species pairs, as the divergence is too high and the mitochondrial Ne too low. Finally, it’s unclear how hemiplasy could lead to the “attraction” or “repulsion” signals observed here.**

- The earlier study benefitted from having time-resolved measurements of genetic changes in influenza. With these data they could detect "directionally epistatic" interactions in historical influenza sequence datasets, suggesting epistatic interactions might be important over the timescales of decades (see below). With the OXPHOS system, we are considering hundreds of thousands of years of divergence. So we sometimes might not be as confident about the timing of different ancestral branch points. Does this affect the analyses in the present study?

**We are not using the timing in our analysis; all that matters is the tree shape. Any phylogenetic analysis will be sensitive to errors in tree reconstruction; however, the ordering of substitutions, which is the basis of our analysis, is rarely ambiguous. We partially account for statistical uncertainty in tree reconstruction (see first section of the Results).**

Further, as the authors pointed out here and in reference 36, by having large genetic distances, mutual information or DCA approaches work well for detecting co-evolving sites because mutation diversity is less affected by phylogeny. How did phylogeny help compared to these approaches? I don't really see how the approach presented in this study has led to different conclusions compared to other approaches.

**Thank you for this question. A detailed comparison between phylogeny-explicit and DCA methods is outside the scope of our study. However, to illustrate a comparison between the methods, we now compare the power of our method to predict contacts in protein structure with that of CCMPred, which is one of the DCA family methods. We find that our method has a sufficient advantage over CCMPred for COX1, which is the most conservative protein encoded in the mitochondrial genome (fig. 1-2 below). This suggests that methods that directly account for the phylogenetic relations between sequences could help for relatively conservative proteins.**

**Fig1. The dependence of precision (aka PPV, positive predictive value = TP/(TP + FP) = 1 - FDR) on the number of pairs selected from top of the list of all pairs ordered by the interaction statistics used by each algorithm. All pairs were ordered separately for each curve. For ccmpred the “confidence” statistics was used, while for our method we used association statistics. Each dot represents PPV for a list of top pairs of length of 10 to 2*L, where L is the protein length. All selected pairs form TP + FP, where TP are those pairs which are in contact (distance < 4A) in 3D structure.**

**Fig2. ROC curves for ccmpred and epistat7.1. The curves and ROC AUC scores were computed using the scikit-learn python package. Site pairs which are in contact (distance < 4A) in 3D structure were defined positive, the others were defined negative.**

- The authors refer to LEGO blocks in the author summary. I love LEGOs, especially when used as an analogy outside the tired context of the "modularity" of cellular signaling and metabolic networks, and so obviously this analogy piqued my interest. Unfortunately, I felt this interesting idea needed to be elaborated on more clearly in this explanatory paragraph as well as in the text.

**We removed “LEGO” from the Authors Summary.**

- Tables. I have never seen tables like this. Most tables appear to be count data across parameter scans, with columns corresponding to arbitrary cutoffs in pre-FDR p-values. Next to each count statistic is an extra "FDR" column, which adds to confusion and makes the tables even more difficult to follow. The counts within a column can differ by orders of magnitude and it isn't clear if this is because there are simply more site pairs for the given protein or whether it is biologically meaningful. For these tables, I encourage the authors to consider whether they are really benefitting their claims, and if so, then to consider showing counts as proportions of total pairs and plotting these values as a function of FDR. The pre-multiple testing p-value is not informative.

**The tables are now thoroughly revised, in accordance with the comments of Reviewers 1 and 3. We now show comparisons of numbers of predictions for the data and for the null model in supplementary figures S1 and S2, and the simulated data, in supplementary figure S3.**

- Figures 1 and 2. It seems these are meant to illustrate how the algorithm works. Not much useful data can be gleaned from the figures, which include thousands of leaves, and comparison between right and left-handed mirror trees is difficult to follow at first. It was much clearer how the algorithm worked in the simpler graphs from earlier publications from these authors (Plos Gen refs 36 and 37).

**These figures are meant to illustrate examples of concordantly and discordantly evolving site pairs. We have now revised them, added an in-figure legend, a cross-referenced them with Figure 3 which indicates where these pairs lie in terms of our statistics. We now also add two figures explaining our method, Fig. 1 and Fig. 2 .**

- Figure 3. Big issue: what is that color statistic?

**As detailed in the caption, the statistic shows the ratio of observed edge densities within and between groups to that expected in the random graph model used by the community detection algorithm [46 ]. We have now added a description of graph building procedures and the formulas used to compute the statistics for Fig. 6 (formerly, Fig. 3): see section “Visualizing contact and coevolution graphs” in Supplementary Methods.**

It's a proportion - why not put it in log scale so that it is proportionally the same for values < 1 as for values > 1?

**Thank you, we are using a log scale now the same for each column.**

Why change the range of values for each panel? Seems there should be a standard scale at least down columns, where the same statistic has been calculated across proteins. But then, indeed, the values vary wildly column-wise and row-wise between panels. Why is this? More explicit discussion of why these values vary so dramatically would help.

**The point we are making with this figure is that the self-connecting edges tend to have high values for contact graphs and positive coevolution graphs, and low values for negative coevolution graphs, and the edges connecting different nodes, vice versa, tend to have high values for negative coevolution graphs and low values for contact graphs and positive coevolution graphs. On the whole, the idea of this figure is to illustrate that groups of coevolving sites tend to be enriched with internal edges both in terms of the 3D structure and positive epistasis. We do not care much about the variety of the scales here. We’ve updated the description of Fig. 6 (formerly, Fig. 3) to clarify the idea of our visualization.**

- Figure 3. Why does cluster 6 in panel (C) or cluster 4 in (J) ... etc ... not connect to itself?

**Thank you for helping us to spot an error. In the right column in figure 3, only edges with negative weights are shown. For the site cluster 6 of the COX1 gene shown in panel c, there were no edges with negative weights that have both vertices in this cluster, so it has no self-connection. The cluster 4 of ATP6 shown in panels j, k and l contains only a single site (see Tab. 5), so it also has no self-connection. Due to a minor error in our scripts, this cluster erroneously had a self-connection in panel k. We have now fixed this error and recomputed the graphs. The difference was minor, but now we don’t have any spurious self-connections.**

- Consider annotating your figures with a bit more text. For example, the panels of Figure 3 are so interrelated by rows and columns such that it is really one figure with different facets. Labelling the rows and columns would help the eye, as well as help understand what statistic is that is being mapped on the figure.

**Thank you for your suggestion. We now include gene names and graph types in our figures for better readability.**

- Similarly, for Figure 4, label the panels with the name of the protein in the structure, and put an axis with a "180º" arrow around it to indicate that rotated the structure is rotated.

**We labeled all panels with gene names.**

**We are not sure that we have understood your question about “rotated structure” correctly. Bovine COX is a homodimer (see PDBID: 1occ). In figure 7 (former figure 4) each protein is presented by two subunits A,N for COX1, B,O for COX2 and C,P for COX3 in their natural positions relative to each other (no rotation used). We have addressed it now in the figure legend. For illustrative purposes all other subunits were hidden. Entire complex was rotated to select the best projection demonstrating the correspondence of sites to coevolving groups.**

- Each of the proteins studied in the work has a crystal structure and the authors report contact graphs for all of them in figure 3. Why not show all the structures as in figure 4, at least in the supplement?

**Figures 3 and 4 (figures 7 and 8 in revised version) show four out of five genes there we observe for some groups significant associations with interfaces with other proteins in corresponding respiratory complexes. For CYTB we do not observe significant associations with protein-protein interfaces (Tab. S9 ) or other evidence for functionality of groups of coevolving sites. Thus for CYTB we have no idea which projection to select to show the protein on the plain. Now, we provide scripts for visualizing structures of all five proteins in JMol (**[**https://sourceforge.net/projects/jmol/files/Jmol/**](https://sourceforge.net/projects/jmol/files/Jmol/)**) as Supplementary Data (results/coevolution_graphs/partitions/pdb/*.rasm). Sites for each protein have been colored according to their assignments into different coevolving groups. Now it is possible to study our results interactively.**

- For the data please use a more standard compression algorithm than '7z'. Your readers shouldn't have to search the internet to download special software to open arcane filetypes to simply see the data.

**Changed to zip.**

------------------------------------
Methods comments
------------------------------------

Line 30: "Evolution in most protein sites is constrained by alleles in other sites, this phenomena is called epistasis."
A set of additive interactions between coevolving sites could give rise to such apparent constraints.

**In the revised version of the manuscript we show that both the concurrent episodic selection and epistasis contribute to the signal in our data. Also, we show that structural proximity of concordantly evolved site pairs are most likely explained by epistasis. Here we would like to focus attention on the epistasis as the most probable reason for observed correlations between coordination or discordance of site evolution and protein structures.**

Line 413: 3 structures are mentioned when structural data for 5 proteins are described in the text. Why only 3 and not 5? The information about the B. taurus alignment is confusing as well. Please rephrase to more explicitly describe what you did there.

**The PDB file 1occ includes structures for all subunits of the respiratory IV complex including three proteins from our study: COX1, COX2 and COX3.**

**We clarified the description of the procedure used to make a correspondence between columns of MSA and sites in protein structures.**

Line 421: "Mutations that followed one another had higher weight" - what was the equation for that? What was the exact exponential penalty? What were the time units for this statistic?

**We add the Supplementary Methods where we formally define our statistics. The time between mutations is measured in units of branch length of the phylogenetic tree.**

Line 422:
"Unlike [36], we did not distinguish between “leading” and “trailing” sites; instead, the epistatic statistic was defined for an unordered pair of sites as the sum of the statistics for the two corresponding ordered pairs."

Why this change? Did the authors find weak evidence for this directional epistasis?

**We have clarified this in the answer to the corresponding question in the “Minor comments” section. The main reason is that for many site pairs substitutions occurred on the same branch.**
