# Supplementary material for: Episodic evolution of coadapted sets of amino acid sites in mitochondrial proteins
Source: PLoS Genet. 2021 Jan 25;17(1):e1008711. doi: 10.1371/journal.pgen.1008711 (PMC7861529; doi:10.1371/journal.pgen.1008711)
Supplement: Attachment — Submitted filename: review.docx [file pgen.1008711.s035.docx]

The authors have responded well to most of my original comments and I would like to see this published as the novel phylogenetic detection of negative epistasis is useful and interesting. However, there are some remaining issues.a

1) I had to work way too hard to understand the section “Concordant and discordant evolution can be caused by episodes of epistatic or additive selection”. This is a crucial section as it shows how epistasis can be distinguished from a confounder, episodic selection. Specifically, language such as “concordance of consecutive substitutions…repulsion of nonconsecutive substitutions” is ill-defined. Repulsion in time or across lineages? Aren’t they already repulsed, being non-consecutive? Or “discordance of consecutive substitutions” - how can consecutive substitutions be in discordance?

The authors’ reasoning is hard to follow. To help, the authors should make sure the understanding that I have for how they disentangled positive epistasis from episodic positive selection is correct or not: Episodic selection alone will have clustering of mutations in two sites across nearby lineages experiencing similar selective forces but with no signal of increased temporal clustering in the same lineage. In contrast, positive selection will have temporal clustering in the same lineage, and may or may not have clustering of mutations across nearby lineages. For negative epistasis, two mutations will be clustered across nearby lineages experiencing similar selective forces but with a lower than expected frequency of the two mutations within the same lineage.

To aid the reader please go by the reasoning for these expectations step by step.

The accompanying Table 1 and figure 2 are also not very helpful, I don’t understand table 1 at all. Because of the unclear language, for example “clustering” (temporally, within same lineage, across different nearby ones?) and “discordance of consecutive substitutions”, etc., figure 2 is confusing.

2) Now that the authors have a method for distinguishing epistasis from episodic selection, I think a better discussion of the limitations of the method to detect epistasis is warranted. In what situations may the method miss epistasis or have some remaining confounders? For example:

-Figure 2, quadrant I is stated in the legend to be a signal of episodic selection, not positive epistasis? But it seems that the pattern could also be due to positive epistasis. Thus, does the method fail to pick this situation up because it cannot distinguish it from episodic selection?

-Let’s say mutation A is selected for in a low temperature environment and mutation B in high temp environment. These mutations will show discordance. But will your method pick this up as negative epistasis? (more generally, any time 2 mutations are positively selected for in different, negatively correlated environments)

3) The section “Concordant or discordant evolution do not result from site-specific episodes of positive

selection or hitchhiking” – please write a couple sentences on the possible or intuitive reasoning why hitchhiking, clonal interference, etc do not cause the observed concordant/discordant patterns your method picks up. Otherwise these analyses are reassuring but do not increase our understanding of the method much.
