# Supplementary material for: Episodic evolution of coadapted sets of amino acid sites in mitochondrial proteins
Source: PLoS Genet. 2021 Jan 25;17(1):e1008711. doi: 10.1371/journal.pgen.1008711 (PMC7861529; doi:10.1371/journal.pgen.1008711)
Supplement: Attachment — Submitted filename: responce_to_rewiers2.docx [file pgen.1008711.s036.docx]

### Reviewer #1:

The authors have properly addressed the reviewers’ comments and substantially improved the manuscript from the last version.

The panel names for Figure 5 should be a and b rather than cd, or Fig 4 and Fig 5 should be merged together.

**Thank you for the comment, now labels for Figure 5 are“a” and “b”.**

### Reviewer #2:

The authors have responded well to most of my original comments and I would like to see this published as the novel phylogenetic detection of negative epistasis is useful and interesting. However, there are some remaining issues.a

1) I had to work way too hard to understand the section “Concordant and discordant evolution can be caused by episodes of epistatic or additive selection”. This is a crucial section as it shows how epistasis can be distinguished from a confounder, episodic selection. Specifically, language such as “concordance of consecutive substitutions…repulsion of nonconsecutive substitutions” is ill-defined. Repulsion in time or across lineages? Aren’t they already repulsed, being non-consecutive? Or “discordance of consecutive substitutions” - how can consecutive substitutions be in discordance?

**Thank you for the comment, now we have unified terminology in this section and through the text. Characterizing patterns of substitutions in a pair of sites on a phylogenetic tree we talk about a concordantly evolving pair of sites, when the excess of rapid consecutive substitutions has been detected in sites of the pair and we talk about a discordantly evolved site pair, when there is a deficit of rapid consecutive substitutions in sites of the pair. Each site pair is also characterized by an average distance between substitutions in different lineages. Now we talk about a phylogenetic clustering or repulsion of substitutions only in the context of whether the distances between nonconsecutive substitutions are less or greater than expected ones.**

The authors’ reasoning is hard to follow. To help, the authors should make sure the understanding that I have for how they disentangled positive epistasis from episodic positive selection is correct or not: Episodic selection alone will have clustering of mutations in two sites across nearby lineages experiencing similar selective forces but with no signal of increased temporal clustering in the same lineage.

**When we talk about episodic selection, we mean that in some lineages or clades the site substitution rate increases or decreases compared to the rest of the tree. If so, your statement is not completely correct, because episodic selection alone would increase the clustering of substitutions in different as well as in same lineages. For a particular site pair, if the episodic selection takes place, we are unable to assert with certainty whether the positive epistasis between sites exists or not.**

In contrast, positive selection will have temporal clustering in the same lineage, and may or may not have clustering of mutations across nearby lineages.

**Probably, here you are rather talking about positive epistasis not about ‘positive selection’; if so, this is a correct statement with the exception that positive epistasis does not cause clustering of substitutions in different lineages in two different sites, because the coefficient of selection in a site is dependent only on whether or not a substitution in another epistatically interacting site is occured.**

**The following statement is correct: “In contrast, positive epistasis will have temporal clustering in the same lineage, and may or may not be accompanied by clustering of mutations across nearby lineages.“**

For negative epistasis, two mutations will be clustered across nearby lineages experiencing similar selective forces but with a lower than expected frequency of the two mutations within the same lineage.

**It is a partially correct statement. Maybe, it could be better formulated in analogy with your previous statement about positive epistasis: In the case of negative epistasis there will be a deficit of consecutive substitutions in the same lineages that may or may not be accompanied by clustering of substitutions in nearby lineages.**

To aid the reader please go by the reasoning for these expectations step by step.

The accompanying Table 1 and figure 2 are also not very helpful, I don’t understand table 1 at all. Because of the unclear language, for example “clustering” (temporally, within same lineage, across different nearby ones?) and “discordance of consecutive substitutions”, etc., figure 2 is confusing.

**We unified terminology now. From the context of the question we could conclude that you are talking about table 2 instead of “Table 1” here. A single reason to include Table 2 in the manuscript was to help with interpretation of correlation coefficients between association statistics and substitution clustering z-scores. Interpretation of a positive correlation coefficient between statistics implies coordinated episodic selection between protein sites and interpretation of a negative correlation coefficient implies epistasis. In the revised text of the manuscript we excluded the former table 2 and added the interpretation of signs of correlation coefficients into the text of the corresponding section of Results.**

2) Now that the authors have a method for distinguishing epistasis from episodic selection, I think a better discussion of the limitations of the method to detect epistasis is warranted. In what situations may the method miss epistasis or have some remaining confounders? For example:

-Figure 2, quadrant I is stated in the legend to be a signal of episodic selection, not positive epistasis? But it seems that the pattern could also be due to positive epistasis. Thus, does the method fail to pick this situation up because it cannot distinguish it from episodic selection?

**Yes, we could not exclude the positive epistasis for the site pairs that fault into the quadrant I on the figure 2, and our method is unable to distinguish it from episodic selection. It seems that concordant episodes of positive selection is the most probable explanation for site pairs that fault into quadrant I and have high values on the ordinate axis. However, for site pairs having low absolute values on the ordinate axis in quadrants I and IV , we showed that probabilities of derived alleles in trailing sites in concordantly evolved pairs are highly dependent on derived alleles in background sites that support the epistatic hypothesis (see section “In a concordantly evolving site pair, allele arising at one site is dependent on the previously arisen allele at the other site”).**

-Let’s say mutation A is selected for in a low temperature environment and mutation B in high temp environment. These mutations will show discordance. But will your method pick this up as negative epistasis? (more generally, any time 2 mutations are positively selected for in different, negatively correlated environments)

**No, this case likely would be classified into the quadrant III for which, again, we are unable to disentangle epistasis and episodic selection. It seems that the scenario of discordant episodes of selection is the most probable explanation for site pairs in quadrant III.**

3) The section “Concordant or discordant evolution do not result from site-specific episodes of positive

selection or hitchhiking” – please write a couple sentences on the possible or intuitive reasoning why hitchhiking, clonal interference, etc do not cause the observed concordant/discordant patterns your method picks up. Otherwise these analyses are reassuring but do not increase our understanding of the method much.

**Thank you for your suggestion. We add the corresponding paragraph into the Discussion.**

**In the new version, we also removed the third simulation for the following reason. Although its results were the same as of the first two (i.e., no spurious signal), we now believe this simulation to be a biologically unrealistic model for the study system, as it is unlikely that the mitochondrial gene trees (and therefore corresponding opisthokontan species trees) are shaped by recurrent advantageous mutations in their mitochondria.**

### Reviewer #3:

I like the new version of the manuscript more and the authors have addressed most of my concerns. I appreciate the effort they put into their response to my comments and find the analysis of comparison between DCA and their method very interesting.

However, I still have concerns about the authors' (quantitative) null prediction for evolution under no epistasis. I do not understand why the selection process of epistasis cannot be modeled to confirm their reasoning about what processes should give rise to observed "repelling" or co-occurrence patterns. Correspondingly, sections (table 2, e.g.) where these hypotheses are discussed do not seem to have much theoretical (analytic or numerical simulation) support; no quantitative, concrete hypothesis of the null distribution of substitutions under epistatic vs non-epistatic selection seems to have been tested. For example, if section beginning line 164 was such a test, why were no epistatic parameters added to illustrate that these were necessary and sufficient to cause repelling/co-occuring patterns of evolution? The lack of these models makes a lot of their reasoning around Line 207 fuzzy to me.

**In response to this, we now model both positive and negative epistasis. We added the new section into Results where we show that positive epistasis indeed increased the number of rapid consecutive substitutions in same lineages and negative epistasis between sites cause the deficit of consecutive substitutions. We also show that association statistics used for aggregating of sites into coevolving groups could be used for ranging of site pairs, so true positively epistatically interacting site pairs are among top pairs with highest positive values of statistics and true negatively epistatically interacting site pairs are among top pairs with highest absolute values of negative statistics.**

Line 56-57: "The main factor affecting the fixation probability is selection favoring some variants over others." Please clarify - the strength of selective pressure relative to mutation rate, demography, and drift are what affect fixation probability

**In response, we changed this fragment as follows: “The fixation probability of a mutation is affected by several factors, namely, changes of a mutation rate, changes of effective population size due to demographic processes or by selection favoring some variants over others”.**

Section beginning line 84. Please define epistasis (it was done briefly in the "author summary" section but must be elaborated in the main text).

**Thank you, we added the definition of epistasis into this paragraph.**

Line 94. Evidence was presented for varying selective pressures in protein evolution. No evidence of epistasis was presented in the paragraph above and so you can't say "These data suggest that the effect of epistasis on the rate of evolution strongly depends on the identity of the interacting partners"

**Yes, we agree that this example rather demonstrates variance of selective forces. We move this evidence into an appropriate place in the Introduction.**

Line 97. This is good evidence of epistasis in these proteins. Is there really nothing else?

**Yes, we have added here references on some interesting examples of mitonuclear epistasis**.

Line 99. First mention of OXPHOS and you have never formally present which proteins you are studying in the text.

**Thank you for the comment, we have clarified it now in the Introduction below:**

**“We apply this method to the evolution of five proteins COX1, COX2, COX3, CYTB and ATP6 encoded in mitochondrial genomes of metazoans and fungi”.**

Line 107. Coevolution can occur in the absence of epistasis and therefore is not evidence of epistasis.

**We clarified it now: “Finally, sites in close proximity in protein structures tend to coevolve most likely due to epistatic interactions among contacting sites”**.

Table 1. # pairs is shown. How many pairs were tested?

**Thank you for the question, now we clarified it in the Methods section.**

**For each protein we considered all possible N*(N-1) unordered pairs of sites, where N is a number of analyzed sites of corresponding multiple alignment. The numbers of analysed sites are provided in the Methods and in the Supplementary Table S10. Also, the total number of pairs those sites are mappable on bovine protein structures are provided in the Supplementary Table S5 (**#all pairs**).**

Line 141: 'Statistic' - please explain what this statistic is, and whether and how this is different from the "association statistic"

**Here we talk about the epistatic statistic that has been previously defined in the paper of Kryazhimsy et. al. 2011, and also it is indicated as “e_(i,j)” in the Supplementary Methods. The raw epistatic statistics are not suitable for comparison between different site pairs, for this reason, we use its z-score transformations normalized on the maximum absolute value of the z-scores across all pairs referred by the term “pseudo-correlations”. However, pseudo-correlations are not independent for different site pairs, e.g., a high value of the pseudo-correlation for a site pair, say (A,C), could be induced by other highly correlated site pairs, say (A,B) and (B,C). To decrease impact on the analyses of such transitive correlations we inferred direct (partial) correlations from pseudo-correlations and referred to them in the manuscript by the term “association statistics” (see sections “Association statistics” in Methods and “Identifying interaction between sites” in Supplementary Methods).**

Line 148: "the test has different power to detect positive and negative associations." Providing an intuition of why this is the case will likely help the reader to understand the tests that have been performed.

**The power of the phylogenetic test to detect concordant and discordant evolution of sites is likely dependent on the numbers of substitutions in these sites. The concordant evolution is easier detectable for a pair of relatively conservative sites, however, the discordant evolution much likely could be detected for pairs of sites with high numbers of substitutions. This intuition follows from the model of independent evolution of protein sites we used. We shuffle substitutions in sites on the phylogenetic tree preserving the numbers of substitutions in each site and on each tree branch. A concordantly evolving site pair is detected if there is a significant excess of rapid consecutive substitutions in these sites that it is very unlikely in the model of independent evolution on large tree, similarly, the discordant evolution which is inferred from the strong deficit of consecutive substitutions is easier to detect for fast evolving sites than for conservative ones.**

**We now shortly explain this intuition in Discussion: “**Our power to detect positive and negative epistasis differs: positive epistasis is more easily detectable, probably because an excess of rare events (pair of consecutive substitutions) is more evident than their deficit”

**This logic is now also supported by simulations, in which as we show our power to detect positive epistasis is higher than that for negative epistasis.**

Line 153: FER vs FDR. Please provide a discussion somewhere in the text on how to interpret the FER as you provided to one of the reviewers.

**Thank you for a question. In the revision we slightly changed the epistatic statistics (see Supplementary Methods). The new statistics, generally, performed better than the previous one, thus it enabled us to use a more stringent threshold (FDR<0.3) for nominal p-values of concordantly and discordantly evolved site pairs. So, we did not use the FER correction for multiple testing in this revision of the manuscript anymore.**

Section line 164: Interesting that no simulation under different demographic scenarios could produce the patterns of substitution you observe in the OXPHOS proteins. Are these methods sufficient to produce any such a signal at all? I.e., what parameters must you change or introduce to reproduce the patterns of substitution you observe in OXPHOS?

**In the revision, we introduce epistatic simulations and show that they give rise to concordance (under positive epistasis) or discordance (under negative epistasis) of substitutions.**

This section would benefit from more explicit statements of hypotheses being tested and ruled out.

Line 207: Clear hypotheses are presented for epistasis of positive or negative nature. However, without clear analytical or numerical demonstration of how these signals would emerge as part of the evolutionary process, it is not clear whether there is a quantitative prediction of the null hypothesis.

**In the revision, we introduce epistatic simulations and show that they give rise to concordance (under positive epistasis) or discordance (under negative epistasis) of substitutions.**

Line 258: "To explain it, one needs to invoke an episode of selection favoring substitutions at both sites individually, but disfavouring their combination – i.e., an episode of negatively epistatic selection." This seems to be a quantitative prediction which must be defended with analytically or numerically.

**The newly added epistatic simulations support this expectation.**

Table 2. Categorization of different types of 'dis/concordant' evolution across episodes of selection are presented. Are the X's and Check Marks in the boxes made by eyeing the data and deciding what fell where? How are there no error or counts here?

**In the former table 2 corresponded to plausible (the check boxes) and unrealistic (X marks) interpretations of observed correlations between association statistics and substitution clustering z-scores. We removed the former table 2 from the text now because it was not very informative.**

line 314. Table S5. Correlation coefficients are significant but small (avg ~0.15). Please state the range of the statistics here and wherever there are mentions of 'significance' pointing to the supplement in the main text so the reader has a sense for the scale of these tests.

**We now have shown the range of Spearman’s correlations in the manuscript.**
